# Health Risk and Pathogenesis of PM_2.5_ in Human Systems

**DOI:** 10.3390/toxics14040286

**Published:** 2026-03-27

**Authors:** Ronghua Zhang, Zhengliang Zhang, Ziru Zhou, Fang Yi, Yulan Yang, Dongmei Guo, Qianying Zhang, Hanyan Wang, Yang Chen, Jingli Qian, Shike Shang, Fumo Yang, Mi Tian, Jingyu Chen, Shumin Zhang

**Affiliations:** 1 Institute of Basic Medicine, North Sichuan Medical College, Nanchong 637000, China; zhangrh202112@163.com (R.Z.); 17729868846@163.com (Z.Z.); ziru_zhou@outlook.com (Z.Z.); yifangmaria@163.com (F.Y.); yulanyang@nsmc.edu.cn (Y.Y.); dongmeiguo@nsmc.edu.cn (D.G.); zhangqianying@nsmc.edu.cn (Q.Z.); wanghanyan@nsmc.edu.cn (H.W.); 2 Department of Stomatology, Shenzhen People’s Hospital Longhua Branch, Shenzhen 518109, China; 3 School of Public Health, North Sichuan Medical College, Nanchong 637000, China; 4 Research Center for Atmospheric Environment, Chongqing Institute of Green and Intelligent Technology, Chinese Academy of Sciences, Chongqing 400714, China; chenyang@cigit.ac.cn; 5 Department of Traditional Chinese Medicine and Rehabilitation, First People’s Hospital of Chongqing Liang Jiang New Area, Chongqing 401121, China; 18523590140@163.com (J.Q.); fyt.g@163.com (S.S.); 6 College of Architecture and Environment, Sichuan University, Chengdu 610065, China; fmyang@scu.edu.cn; 7 Key Laboratory of Three Gorges Reservoir Region’s Eco-Environment, Ministry of Education, Chongqing University, Chongqing 400045, China; tianmi628@cqu.edu.cn

**Keywords:** PM_2.5_, multi-system toxicity, molecular mechanisms, health risk

## Abstract

Fine particulate matter (PM_2.5_) poses a significant global environmental health threat and is closely associated with diseases across multiple organ systems. This review systematically summarizes the toxic effects and underlying mechanisms of PM_2.5_ in the respiratory, cardiovascular, nervous, immune, endocrine, digestive, and genitourinary systems. Key pathogenic processes involve shared pathways such as oxidative stress, inflammatory responses, endoplasmic reticulum stress, autophagy, and apoptosis, along with the activation of system-specific signaling networks. The complex composition and notable spatiotemporal variability of PM_2.5_ present challenges for assessing its health risks and clarifying its mechanisms. Moving forward, integrating multi-omics and molecular epidemiology approaches will be essential to unravel its multi-system pathogenic networks and support the development of effective intervention strategies.

## 1. Introduction

Air pollution, driven by global industrialization and urbanization, poses a critical threat to ecological systems and human health. PM_2.5_ is especially concerning due to its well-documented role in provoking systemic health impairments. In response to growing evidence of harm even at low exposure levels, the World Health Organization (WHO) updated its Global Air Quality Guidelines in September 2021, lowering the recommended annual PM_2.5_ limit from 10 μg/m^3^ to 5 μg/m^3^ [1]. This revision underscores the urgency of mitigating PM_2.5_ pollution worldwide. Additionally, studies have also found that approximately 90% of the global population resides in areas where air quality fails to meet WHO standard [2], underscoring the ongoing health risks associated with PM_2.5_ exposure. Research further reveals that PM_2.5_ significantly elevates the incidence and mortality rates of respiratory and cardiovascular diseases, as well as causing systemic disorders such as neurodegenerative, immunocompromised, endocrine-disrupting, urogenital, and other multi-organ system pathologies [3]. However, much of the existing literature reviews focus on single-organ or disease-specific outcomes, leaving a gap in synthesized, multi-system pathophysiological understanding. To address this, the present review comprehensively examines the mechanisms of PM_2.5_ toxicity across all major organ systems, emphasizing common pathways such as oxidative stress and inflammatory signaling, while also highlighting system-specific pathological processes. By integrating current evidence across disciplines, this work aims to support more holistic public health strategies and informed regulatory responses to ambient PM_2.5_ pollution.

## 2. Search Strategy and Study Selection

### 2.1. Search Strategy

We systematically searched PubMed, ScienceDirect, Google Scholar, Baidu Scholar, and CNKI for studies published between January 2000 and February 2025. The search combined terms for PM_2.5_ (“particulate matter”, “PM_2.5_”), organ systems (respiratory, cardiovascular, nervous, immune, endocrine, digestive, genitourinary), and outcomes (“toxicity”, “pathogenesis”, “mechanism”, “health effect”). For each specific disease, additional searches were performed using combinations of “PM_2.5_” with the disease name and relevant mechanism terms (e.g., “apoptosis”, “inflammation”), or directly with the disease name alone. The search was limited to English- and Chinese-language publications.

### 2.2. Inclusion and Exclusion Criteria

Studies were included if they: (1) addressed PM_2.5_ exposure in relation to any of the seven organ systems; (2) were original research (human epidemiological studies, animal experiments, or in vitro studies) or systematic reviews; (3) provided data on health outcomes or mechanisms. We excluded non-original articles (e.g., editorials, conference abstracts), duplicate publications, and studies without full text availability. The detailed numbers of records identified, deduplicated, screened, and finally included for each organ system are provided in Supplementary Table S4.

### 2.3. Geographic Scope

No geographic restrictions were applied. The predominance of Asian studies reflects higher research output in regions with elevated PM_2.5_ concentrations; we have included representative studies from other regions to maintain a balanced global perspective.

### 2.4. Exposure Assessment Considerations

The studies reviewed employ different PM_2.5_ exposure measures. Most epidemiological studies use ambient concentrations from fixed-site monitors, which serve as population exposure proxies but may misclassify individual exposure due to unaccounted factors (e.g., time-activity patterns, indoor infiltration). A subset of studies, particularly from Asia, use personal exposure monitoring or model indoor concentrations based on outdoor levels and infiltration factors. Experimental studies (animal, in vitro) typically use controlled exposure to concentrated ambient particles or specific components (e.g., diesel exhaust particles). Unless otherwise specified, “PM_2.5_ exposure” in this review refers to ambient air pollution, consistent with most cited literature. 

## 3. Definition of PM_2.5_

PM_2.5_ is defined as fine particles with an aerodynamic diameter of ≤2.5 μm [4,5]. Owing to their small particle size and high specific surface area, PM_2.5_ particles can remain suspended in the atmosphere for extended periods and undergo long-range transport, contributing to transregional pollution and human exposure far from emission sources [6]. Their composition is complex and heterogeneous, encompassing sulfates, nitrates, organic compounds, heavy metals, and various other toxicants, which collectively underlie their adverse health effects [7]. Due to these properties, PM_2.5_ not only impairs atmospheric visibility but also poses significant risks to human health. Upon inhalation, PM_2.5_ can penetrate deep into the lower respiratory tract and alveolar regions, cross the alveolar-capillary barrier, and enter the systemic circulation [8]. Consequently, it can initiate multiple injury mechanisms and cause damage to various organ systems throughout the body.

## 4. Chemical Composition and Health Risks of PM_2.5_

PM_2.5_ is a complex mixture primarily composed of water-soluble ions, organic matter, carbonaceous components, and inorganic elements. Its chemical composition displays significant seasonal variations and distinct urban–rural disparities, which in turn are closely associated with the pathogenesis of various human diseases [9,10,11]. First, water-soluble inorganic ions, particularly secondary ions such as SO_4_^2−^, NH_4_^+^, NO_3_^−^, etc., constitute the most abundant components of PM_2.5_. Their concentrations peak in autumn and winter and have been closely associated with respiratory diseases and metabolic disorders [12,13,14]. Secondly, organic compounds in PM_2.5_ are of concern due to their potential mutagenic, teratogenic, and carcinogenic effects. Polycyclic aromatic hydrocarbons (PAHs), with benzopyrene as a key marker, exhibit significant seasonal variation. Studies in Chinese cities such as Beijing and Taiyuan have reported substantially higher PAH concentrations during cold seasons compared to warm periods [15,16,17]. Furthermore, carbonaceous components primarily encompass organic carbon and elemental carbon. OC has both primary emission and secondary formation sources, while EC is a primary combustion tracer. Studies have found that a high OC/EC ratio is frequently used as an indicator of substantial secondary organic aerosol formation, potentially posing a significant health risk to humans [18]. Additionally, inorganic elements comprised the smallest fraction of PM_2.5_, yet they possess genotoxic and carcinogenic properties, such as Pb, As, Mn, Co and Cr [19,20]. In summary, the chemical composition of PM_2.5_ is closely linked to various health outcomes, including cancer, teratogenesis, inflammation, and mutagenesis.

## 5. Harm of PM_2.5_ to Human Body Systems

**Figure 1:**
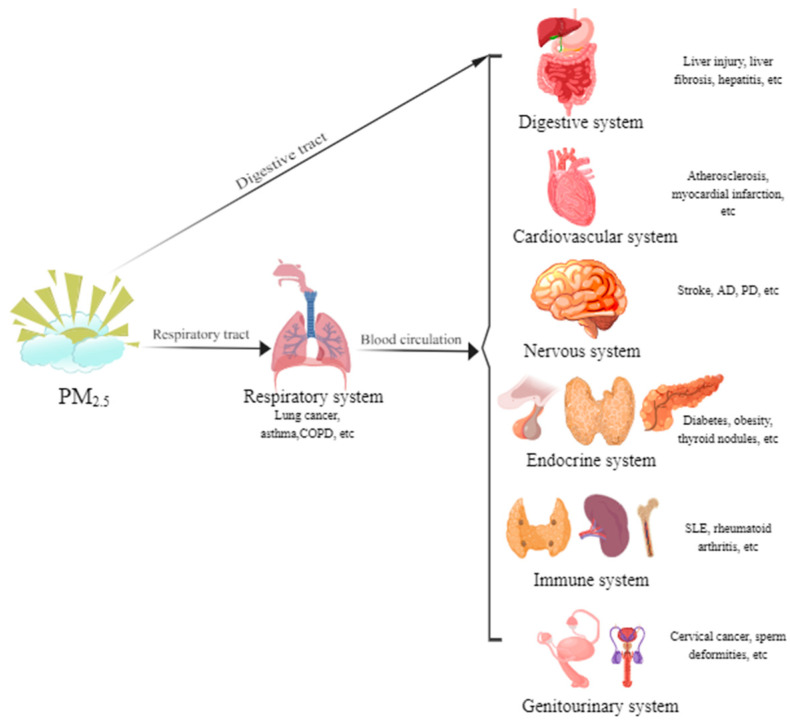
PM_2.5_ and human body systems.

The health impact of particulate matter (PM) is size-dependent, with finer particles posing greater risks due to their ability to penetrate deeper into the respiratory tract. PM_2.5_ is considered one of the most harmful pollutants to human health. Its small size allows it to bypass upper airway defenses, deposit in the terminal bronchioles and alveoli, and even translocate into systemic circulation, thereby causing direct pulmonary injury and multi-organ damage [8] (Figure 1). Therefore, the health effects of PM_2.5_ are of great concern to the scientific community and the public.

As summarized in Table 1, the principal pathogenic mechanisms include oxidative stress, inflammatory response, apoptosis, autophagy, and other contributing factors. PM_2.5_ orchestrates its toxic effects primarily by dysregulating key signaling pathways, with PI3K/Akt, NF-κB, and endoplasmic reticulum (ER) stress emerging as central molecular hubs across multiple organ systems. PI3K/Akt Pathway: This crucial regulator of cell growth and survival is frequently activated by PM_2.5_, driving oxidative stress notably in the respiratory, cardiovascular, nervous, immune, and genitourinary systems [21]. NF-κB Pathway: As a master regulator of inflammation, the NF-κB pathway is extensively exploited by PM_2.5_ to induce inflammatory responses in the respiratory, cardiovascular, nervous, immune, endocrine, digestive, and genitourinary systems [22]. Endoplasmic Reticulum Stress: While initially protective, sustained ER stress disrupts cellular homeostasis and triggers apoptosis [23]. PM_2.5_ induces apoptosis via this mechanism, contributing to diseases in the cardiovascular, nervous, immune, endocrine, and genitourinary systems.

In addition to these common pathways, PM_2.5_ also elicits system-specific effects by targeting distinct mechanisms. In the Cardiovascular System, PM_2.5_ induces dysfunction in the coagulation and fibrinolytic systems, thereby promoting a prothrombotic state which accelerates the pathogenesis of thrombosis and atherosclerosis [75]. Disruption of intracellular calcium (Ca^2+^) homeostasis is a pivotal mechanism underpinning the toxicity of PM_2.5_ across multiple organ systems, albeit with system-specific pathways. In the cardiovascular and respiratory systems, this dysregulation is predominantly driven by ROS-dependent signaling, which leads to abnormal Ca^2+^ flux and subsequent cellular dysfunction. Within the genitourinary system, PM_2.5_ also induces Ca^2+^ overload, which is closely associated with structural and functional tissue injury, highlighting a direct cytotoxic role of calcium imbalance in reproductive and urinary pathologies.

Therefore, PM_2.5_ induces multi-system diseases through a network of shared and system-specific mechanisms. This complexity arises from variations in its source, chemical composition, and size distribution, which influence the spectrum and pathogenesis of resulting diseases. We will discuss the PM_2.5_-related disease types and pathogenesis on the seven major human body systems: respiratory, cardiovascular, nervous, immune, endocrine, digestive and genitourinary systems.

### 5.1. Respiratory System

PM_2.5_ exposure is a major risk factor for respiratory diseases, including lung cancer, chronic obstructive pulmonary disease (COPD), asthma, bronchitis, and pulmonary injury [79] (Table 2 and Table S2A). Epidemiological evidence indicates that each 10 µg/m^3^ increase in PM_2.5_ concentration is associated with a 0.58% rise in long-term respiratory mortality, alongside short-term increases of 2.07% in morbidity and 8% in hospitalization rates, with infants, the elderly, and pregnant women being particularly susceptible [42].

The pathophysiological mechanisms underpinning these effects are multifaceted and interlinked, primarily involving oxidative stress, inflammatory response, autophagy, apoptosis and genotoxicity (Table 1 and Table S3). Following inhalation and deposition in the lungs, PM_2.5_ particles are phagocytosed by alveolar macrophages, leading to excessive production of reactive oxygen species (ROS). This oxidative stress activates transcription factors such as NF-κB, which upregulate pro-inflammatory cytokines and chemokines. Sustained inflammation promotes airway remodeling, exacerbates asthma and COPD, and impairs lungfunction [24,109]. Concurrently, genotoxic components adsorbed on PM_2.5_—notably polycyclic aromatic hydrocarbons (PAHs) and heavy metals—can directly induce DNA strand breaks, form DNA adducts, and cause chromosomal aberrations and micronucleus formation. These genetic alterations drive mutagenesis and carcinogenic transformation, significantly elevating the risk of lung cancer [24,109]. In addition, PM_2.5_ can disrupt cellular homeostasis by activating the PI3K/AKT/mTOR signaling pathway, thereby inducing autophagy and promoting apoptosis in bronchial epithelial cells, which further contributes to pulmonary tissue damage and functional decline [55]. Collectively, oxidative stress-triggered inflammation, direct DNA damage, and dysregulated autophagy–apoptosis pathways act synergistically to form the pathophysiological basis of PM_2.5_-induced respiratory disorders, spanning from chronic inflammatory diseases to lung cancer.

#### 5.1.1. PM_2.5_ and Lung Cancer

According to GLOBOCAN 2020 estimates, lung cancer is the second most prevalent cancer and the leading cause of cancer mortality globally, with the highest burden observed in China [110]. This substantial disease load underscores the importance of identifying and intervening in modifiable risk factors, among which PM_2.5_ stands out as a critical environmental driver. A meta-analysis by Huang et al. reported that A 10 μg/m^3^ increment in PM_2.5_ exposure was linked to a pooled relative risk of 1.11 (95% CI: 1.05–1.18) for lung cancer mortality and 1.08 (95% CI: 1.03–1.12) for lung cancer incidence [111]. Accumulating evidence indicates that PM_2.5_ promotes lung carcinogenesis through a network of interconnected signaling pathways (Table 2 and Table S2A). These include: (1) the enhancement of invasive and metastatic potential through pathways such as ARNT2/PP2A/STAT3/MMP2; (2) the stimulation of aberrant cell proliferation, often mediated by exosome-transmitted signals like Wnt3a/β-catenin; and (3) the fostering of a pro-tumorigenic microenvironment via IL-17a-associated inflammatory signaling, which promotes both tumor progression and immunosuppression [80,81,82]. Moreover, Furthermore, PM_2.5_ upregulates the long non-coding RNA loc146880 by inducing ROS generation, thereby enhancing autophagy and promoting cell migration [83]. Together, these mechanisms illustrate how PM_2.5_ orchestrates a multi-faceted pro-tumorigenic network, driving lung cancer initiation and progression through oxidative stress, aberrant proliferation, invasive capacity, and immune–inflammatory dysregulation.

#### 5.1.2. PM_2.5_ and Chronic Obstructive Pulmonary Disease (COPD)

Chronic obstructive pulmonary disease (COPD) is a progressive inflammatory airway disorder characterized by persistent airflow limitation. Exposure to PM_2.5_ has emerged as a major environmental risk factor for COPD pathogenesis. Epidemiological data from the Global Burden of Disease Study reveal a striking increase of over 90% in both PM_2.5_-attributable COPD deaths and disability-adjusted life years (DALYs) from 1990 to 2019, with the highest burden concentrated in China and India [112]. This surge highlights PM_2.5_ as a critical driver of the global COPD epidemic. Mechanistically, PM_2.5_ drives airway remodeling by activating the Wnt5a/β-catenin pathway, leading to human bronchial smooth muscle cell proliferation [85]. It also amplifies inflammation through microRNAs such as miR-149-5p, which targets TAB2 to modulate MAPK/NF-κB signaling in bronchial epithelial cells [86,87,88]. Furthermore, PM_2.5_ disrupts autophagy by inhibiting the PI3K/Akt/mTOR pathway to induce alveolar epithelial cell apoptosis, and by upregulating NEAT1 to activate PINK1/Parkin-mediated mitophagy, thereby compromising mitochondrial function [89,90]. Collectively, these mechanisms—driving remodeling, inflammation, and alveolar destruction—form a multi-target network that orchestrates the complex pathophysiology of COPD following PM_2.5_ exposure (Table 2 and Table S2A).

#### 5.1.3. PM_2.5_ and Asthma

As a chronic airway disorder, asthma arises from complex gene–environment interactions, where exposure to air pollutants like PM_2.5_ plays a critical role. PM_2.5_ promotes asthma by targeting two key defensive systems (Table 2 and Table S2A). First, it disrupts the airway epithelial barrier through oxidative stress and activation of NF-κB and MAPK signaling, resulting in inflammation and enhanced allergen sensitization [92]. Second, it induces immunotoxicity by disturbing the balance between T helper 17 (Th17) and regulatory T (Treg) cells. This is achieved by activating the STAT3/RORγt pathway to promote Th17 responses, while inhibiting the STAT5/Foxp3 pathway to impair Treg-mediated tolerance [94]. In summary, PM_2.5_ undermines respiratory health by compromising the physical barrier and disrupting the immunological balance in the airways. This dual assault on structural integrity and immune tolerance provides a coherent mechanistic framework for how PM_2.5_ exposure facilitates asthma onset and exacerbation.

### 5.2. Cardiovascular System

Cardiovascular disease (CVD) is the leading cause of global mortality [113], with PM_2.5_ established as a major environmental risk factor. PM_2.5_ contributes to a spectrum of CVDs, including atherosclerosis, myocardial infarction, and acute coronary syndrome (Table 3 and Table S2B). Epidemiological studies distinguish between acute and chronic effects: short-term exposure elevates the relative risk of acute events by 1–3%, whereas long-term exposure is associated with a more substantial ~10% increase in chronic CVD risk [114].

The molecular mechanism of the pathogenicity of PM_2.5_ mainly involves four aspects: oxidative stress, inflammation, apoptosis, autophagy and others (e.g., systemic coagulation abnormalities and Ca^2+^ homeostasis imbalance) (Table 1 and Table S3). Among these, oxidative stress is regarded as a central initiating event. PM_2.5_ exposure promotes excessive generation of reactive oxygen species (ROS), which in turn activates the protective Nrf2 pathway in an attempt to counteract cellular damage, as demonstrated in vascular injury models [25,26]. Interestingly, while PM_2.5_-induced oxidative stress appears to elevate homocysteine (Hcy) levels, and Hcy has been implicated in promoting cardiovascular pathology through pro-oxidative and pro-inflammatory mechanisms [115,116,117,118], direct evidence for this specific cascade as a primary mediator of PM_2.5_ cardiovascular toxicity remains limited. This knowledge gap underscores the need for further mechanistic studies to elucidate whether Hcy serves as a downstream biomarker or an active mediator in PM_2.5_-induced cardiovascular disease. The inflammatory response is another major mechanism, characterized by the activation of the NLRP3 inflammasome, dysregulation of the AKT/eNOS/NO signaling axis, and induction of the TRAF6/NF-κB pathway, among others [27,28,45,46]. In terms of apoptosis, PM_2.5_ can trigger programmed cell death through oxidative stress-mediated pathways involving ROS-Ryr2-Ca^2+^ imbalance, as well as via direct induction of endoplasmic reticulum stress (ERS) and activation of the p53–Bax–caspase cascade [58,59,119]. Regarding autophagy, PM_2.5_ exposure initiates endoplasmic reticulum stress and alters the expression of autophagy-related proteins such as LC3 and p62. Importantly, it often impairs autophagic flux, leading to defective autophagy that may ultimately promote cellular injury [58,120]. Additionally, PM_2.5_ exerts notable effects on the blood system, including platelet activation and a shift toward a hypercoagulable state, which further exacerbates cardiovascular dysfunction [68,120]. In summary, PM_2.5_ induces cardiovascular toxicity through an interlinked series of events: oxidative stress initiates inflammation, which together drive cellular dysfunction and death, autophagy impairment, and systemic coagulation abnormalities. This multifaceted assault culminates in endothelial dysfunction, adverse vascular remodeling, and the clinical manifestations of CVD.

**Table 3:** Pathogenic mechanisms and evidence levels of PM_2.5_-induced cardiovascular diseases.

Disease Type	Target/Pathway	Function	Evidence Level
Atherosclerosis	PI3K/Akt/mTOR [121]	Autophagy	▲ + ■
	NOX2 [122]	Oxidative stress	▲
	Wnt5a/Ror2 [123]	PVAT inflammation	▲
	MAPK [124]	Atherosclerosis	▲
	JAK2/STAT3 [125]	Inflammatory responses and lipid accumulation	■
	IL-6/gp130/STAT3 [126]	Inflammatory responses	R
	NLRP3 inflammasome [127]	Inflammatory responses and Endothelial cell dysfunction	▲ + ■
	TLR2/TLR4/NF-κB and p38/MAPK [128,129]	Inflammatory responses and Oxidative stress	R
Myocardial infarction	MG53 [130]	Cell membrane repair	▲
	JNK/p53 [29]	Inflammatory responses, Oxidative stress and Apoptosis	▲
	CD69^+^Treg cells, miR-146a-5p and miR-423-3p [131]	Immune response and Inflammatory response	★★
	mitochondrial dysfunction [132]	mitochondrial dysfunction	▲
Arteriosclerotic heart disease	Time series study [133]	Epidemiology	★
Acute coronary syndrome	NO, ET-1 and mitochondria damages [134]	Oxidative stress, vascular tone, vasoconstriction and mitochondria damages	R
Ischemic heart disease	β2AR/PI3K/Akt [135]	Apoptosis	▲ + ■
	Oxidative stress and Inflammatory responses [30]	Oxidative stress, Inflammatory responses	R
	PERK/Sestrin2 [136]	Apoptosis and Autophagy	■
	NCOA4 [137]	Ferritinophagy	▲ + ■
Atherosclerotic cardiovascular disease	PERK/Sestrin2 [136]	Apoptosis and Autophagy	■
Heart failure	Oxidative stress and Inflammatory responses [30]	Oxidative stress and Inflammatory responses	R
	NCOA4 [137]	Ferritinophagy	▲ + ■

Evidence levels: ★★ = moderate human evidence (limited human studies or inconsistent results); ★ = weak human evidence; ▲ = animal studies; ■ = in vitro studies. Combined symbols indicate multiple lines of evidence. (R) indicates the mechanism is derived from a review article.

#### 5.2.1. PM_2.5_ and Atherosclerosis

Atherosclerosis is one of the most common cardiovascular diseases, primarily attributable to endothelial dysfunction. Accumulating evidence identifies PM_2.5_ as a significant environmental risk factor that accelerates atherosclerotic progression through multiple interconnected pathways, including endothelial impairment, dysregulated lipid metabolism, and systemic coagulation abnormalities [138]. Endothelial dysfunction is largely mediated by oxidative stress and inflammatory activation. Key signaling pathways involved include NOX2, IL-6/gp130/STAT3, NF-κB, the NLRP3 inflammasome, and Nrf2 [126,127,139]. In parallel, PM_2.5_ exposure disrupts lipid metabolism and promotes foam cell formation by inducing oxidative stress via p38 MAPK and activating inflammation through TLR2/TLR4 signaling [138]. Furthermore, abnormal activation of the coagulation-fibrinolysis system is a critical step in the development of coagulation disorders [75]. Therefore, by concurrently impairing endothelial function, dysregulating lipid metabolism, and inducing a prothrombotic state, PM_2.5_ orchestrates a multi-faceted assault that directly promotes the initiation and progression of atherosclerosis (Table 3 and Table S2B).

#### 5.2.2. PM_2.5_ and Myocardial Infarction

Myocardial infarction (MI) is a leading cause of global mortality and disability, posing a severe threat to public health [130]. Clinical evidence indicates that exposure to PM_2.5_ is not only associated with an increased risk of MI but may also exacerbate disease progression by interfering with myocardial repair mechanisms [140]. A nationwide Spanish study of 115,071 acute MI patients reported that a 3-day average PM_2.5_ concentration exceeding 10 μg/m^3^ was associated with 21.9 additional hospitalizations per 1000 (95% CI: 9.1–34.8; *p* < 0.001), while concentrations above 25 μg/m^3^ increased in-hospital mortality by 14% (OR = 1.14; 95% CI: 1.07–1.23; *p* < 0.001) [141]. Experimental studies provide mechanistic insights into this phenomenon. In rats with established MI, PM_2.5_ exposure was found to downregulate the expression of MG53, a key protein essential for cardiac membrane repair. This impairment of the intrinsic repair capacity likely contributes to the observed aggravation of myocardial injury and the worsening of cardiac function following infarction [130]. Further animal studies have revealed that in hyperlipidemic rats, PM_2.5_ exposure activates the JNK/p53 signaling pathway, promoting cardiomyocyte apoptosis and thereby exacerbating myocardial injury, which models an elevated risk of MI [29]. Furthermore, regulatory T cells, miR-146a-5p, and miR-423-3p are implicated in the pathogenesis of ST-segment elevation myocardial infarction (STEMI) induced by short-term PM_2.5_ exposure [131]. In summary, PM_2.5_ contributes to the initiation and aggravation of myocardial infarction through a multifaceted pathogenesis, concurrently disrupting structural repair, activating cell death pathways, and dysregulating immune and epigenetic homeostasis (Table 3 and Table S2B).

### 5.3. Nervous System

As the central regulatory hub of human physiological functions, the nervous system has been increasingly implicated in the adverse effects of atmospheric PM_2.5_ exposure. Studies indicate that PM_2.5_ can infiltrate the central nervous system via multiple pathways, including translocation across the blood–brain barrier, the pulmonary alveolar–blood barrier, migration along the olfactory nerve pathway, or disruption of the gut–microbiota–brain axis, thereby directly inducing neurotoxicity [142]. At the structural level, PM_2.5_ directly compromises the central nervous system by disrupting the blood–brain barrier. Studies have demonstrated that diesel exhaust particles (DEPs) can directly disrupt tight junctions in cerebral vascular endothelial cells by inducing inflammation, oxidative stress, and activation of the RhoA/ROCK signaling pathway, thereby increasing BBB permeability [143]. The blood–brain barrier (BBB) regulates peripheral–central crosstalk, protecting the central nervous system from blood-borne substances [144]. As a core component of the neurovascular unit (NVU), BBB disruption has consequences beyond increased permeability [142,144]. Breakdown of the BBB permits inflammatory factors and toxins to infiltrate the brain parenchyma, triggering cellular responses that compromise NVU function [142]. A key feature of the BBB is the formation of tight junctions (TJs) by endothelial cells; PM_2.5_ exposure has been shown to disrupt these TJs and concurrently promote glutamate release from macrophages and microglia, leading to reduced neuronal survival [145]. Thus, the BBB represents a critical node through which PM_2.5_ triggers NVU dysfunction and neuronal injury [143]. Studies have demonstrated an association between PM_2.5_ exposure and elevated plasma homocysteine (Hcy) levels [118,146]. Furthermore, a mechanism proposed in non-PM_2.5_ contexts suggests that Hcy may activate endothelial N-methyl-D-aspartate receptors (NMDAr), leading to β-catenin nuclear translocation and subsequent suppression of claudin-5 expression, thereby compromising blood–brain barrier (BBB) integrity [147]. However, as this pathway has not been directly validated in the context of PM_2.5_ exposure, it remains speculative and requires further investigation. Epidemiological evidence further confirms that short-term exposure to PM_2.5_ is positively correlated with hospitalization risks for various neurological disorders, such as ischemic and hemorrhagic stroke, Alzheimer’s disease, Parkinson’s disease, and neurodevelopmental impairments [148] (Table 4 and Table S2C). The neurotoxicity of PM_2.5_ is orchestrated by multiple, interconnected molecular mechanisms. These include: (1) oxidative stress, primarily mediated by ROS generated via various pathways; (2) neuroinflammation driven by the activation of signaling cascades such as NF-κB, JAK2/STAT3, and MAPK; (3) apoptosis involving mediators like p53 and SAPK; (4) dysregulated autophagy modulated by the AMPK/mTOR axis; and (5) epigenetic alterations, such as changes in DNA methylation patterns influenced by DNMT1. The interplay among these processes—where oxidative stress and inflammation often initiate cellular damage, which then engages cell death pathways and epigenetic reprogramming—synergistically contributes to neuronal injury [60,61,142] (Table 1 and Table S3). In summary, current evidence delineates PM_2.5_ as a systemic neurotoxicant, whose impact is mediated through direct infiltration, diverse molecular insults, and their interplay, ultimately elevating the population burden of neurological disorders.

As well, distinguishing the effects of PM_2.5_ from those of co-pollutants (e.g., NO_2_, O_3_) remains a key methodological challenge. Current evidence on air pollution neurotoxicity is largely derived from epidemiological studies [149,150,151,152], while experimental studies isolating individual pollutants are limited. Although most toxicological research has focused on single pollutants, emerging evidence suggests that co-exposure may exert synergistic effects. For instance, PM_2.5_ and O_3_ co-exposure exacerbates neurodegenerative alterations in animal models [149], and systematic reviews highlight complex pollutant interactions [150]. Due to high correlations between co-pollutants, disentangling their independent effects is difficult. Recent studies have addressed this using multi-pollutant models (e.g., single- and two-pollutant models [151]) and stratified analyses [152] to better estimate individual pollutant effects. Further methodological refinements are needed to better isolate PM_2.5_ effects from other pollutants, particularly for neurological outcomes.

**Table 4:** Pathogenic mechanisms and evidence levels of PM_2.5_-induced Nervous diseases.

Disease Type	Target/Pathway	Function	Evidence Level
Stroke (ischemic and hemorrhagic)	ROS, NLR3P3 [153]	Oxidative stress, Inflammatory response, and pyroptosis	■
	NOX/Akt/eNOS/NO [27]	Oxidative stress, Inflammatory damage	■
	Nrf 2/HO-1, NF-κB/TNF-α [154]	Oxidative stress, Inflammatory response and Apoptosis	■
	COX-2/PGES/PGE2, ERK/AKT/NF-κB [155]	Endothelial damage	R
	PI3K/AKT/NF-κB [155]	Inflammatory response	R
Alzheimer’s disease	ROS, PI3K/Akt/FoxO1 [142]	Oxidative stress	R
	AMPK/mTOR [142]	Autophagy	
	PKA/CREB/BDNF [142]	Neuroprotective effects	
	NF-κB [142], NLRP3 [49]	Inflammatory response	
Neurodevelopmental disorders	Mitochondrial damage [156]	Mitochondrial damage	▲
	NF-κB, TNF-α, IL-1β [156]	Inflammatory response	▲
	Caspase family proteins [156]	Apoptosis	▲
	SHANK3 [157]	DNA methylation	▲
Parkinson’s disease	PI3K/Akt/FoxO1 [142]	Oxidative stress	R
	NF-κB [142]	Inflammatory response	
	AMPK/mTOR [142]	Autophagy	
	PKA/CREB/BDNF [142]	Neuroprotective effects	
Dementia	PI3K/Akt/FoxO1 [142]	Oxidative stress	R
	NF-κB [142]	Inflammatory response	
	AMPK/mTOR [142]	Autophagy	
	PKA/CREB/BDNF [142]	Neuroprotective effects	
Schizophrenia	The striatum [158]	Oxidative stress, Inflammation, Astrocyte activation and modifications in dopamine	▲ + ■
Brain tumor	Epidemiology [142]	Oxidative stress and Inflammatory response	R

Evidence levels: ▲ = animal studies; ■ = in vitro studies. Combined symbols indicate multiple lines of evidence. (R) indicates the mechanism is derived from a review article.

#### 5.3.1. PM_2.5_ and Stroke

Stroke is a major cerebrovascular disease, comprising ischemic and hemorrhagic subtypes. Epidemiological studies show that each 10 µg/m^3^ increase in PM_2.5_ is associated with increase in risk of stroke incidence (0.37%) and mortality (0.71%), with ischemic stroke showing stronger associations (0.46% and 1.09%, respectively) than hemorrhagic stroke [159]. Mechanistically, PM_2.5_ is implicated in the pathogenesis of ischemic stroke by inducing oxidative stress and systemic inflammation. This may involve activation of pathways such as NADPH oxidase (NOX)–Akt/eNOS/NO, leading to upregulation of the NLRP3 inflammasome and MMP-9, thereby causing vascular injury and promoting atherosclerosis progression [27]. Furthermore, PM_2.5_ may aggravate ischemic injury through NLRP3 inflammasome activation and pyroptosis [153]. Collectively, these findings highlight the dual role of PM_2.5_ exposure in both promoting stroke onset and aggravating secondary brain injury, primarily through driving systemic oxidative stress and neuroinflammation. This deepens our mechanistic understanding of how environmental pollution influences cerebrovascular health (Table 4 and Table S2C).

#### 5.3.2. PM_2.5_ and Brain Nerve Damage

PM_2.5_ is a recognized neurotoxicant capable of damaging the central nervous system and contributing to various neurological disorders [160]. Epidemiological and experimental studies have linked PM_2.5_ exposure to an increased risk of both neurodegenerative diseases, such as Alzheimer’s disease (AD) and Parkinson’s disease (PD), and neurodevelopmental disorders like autism spectrum disorder (ASD) (Table 4 and Table S2C) [142,161]. Long-term exposure is particularly associated with cognitive decline in the elderly [162,163]. In AD, a progressive disorder primarily characterized by cognitive impairment, PM_2.5_ is shown to exacerbate pathology by inducing neuroinflammation, notably through activating the NLRP3 inflammasome in microglia [49,164]. Gestational and early-life exposure to PM_2.5_ is increasingly recognized as a risk factor for autism spectrum disorder (ASD), with mechanisms operating through multiple exposure windows and pathways. Postnatal direct exposure induces epigenetic downregulation of the synaptic gene SHANK3, disrupting neural connectivity [157]. Prenatal exposure via maternal inhalation programs hippocampal pathologies—including neuroinflammation and apoptosis—in offspring. Placental-mediated pathways operate through two parallel mechanisms [156,165]. First, PM_2.5_ inhalation during pregnancy induces placental hypoxia (evidenced by HIF upregulation), establishing a pathophysiological basis for ASD risk [166]. Second, PM_2.5_-bound polycyclic aromatic hydrocarbons (PAHs) cross the placental and immature blood–brain barriers to accumulate in fetal brain tissue, where they target HSP90AA1, disrupt protein homeostasis, trigger the unfolded protein response, and induce neuroinflammation—leading to aberrant white matter connectivity, a hallmark of ASD pathology [167]. Co-pollutant synergy further amplifies risk: co-exposure to PM_2.5_ and formaldehyde enhances ASD phenotypes at doses where individual exposures show no effect [168].

### 5.4. Immune System

The immune system is a sophisticated yet vulnerable defense network, tasked with surveillance and homeostasis. It represents a primary target for environmental agents (e.g., PM_2.5_), whose impact can subvert its normal function, leading to tissue damage and dysregulation [62,169]. Epidemiological studies estimate that environmental factors account for 40–70% of autoimmune disease etiology, with air pollution implicated as a major contributor [33]. Exposure to PM_2.5_ has been associated with the incidence or exacerbation of several immune-mediated disorders, such as systemic lupus erythematosus, rheumatoid arthritis, and multiple sclerosis (Table 5 and Table S2D).

The immunotoxicity is mediated through multiple mechanisms. Firstly, by inducing oxidative stress, PM_2.5_ can activate pivotal pro-inflammatory signaling pathways, most notably NF-κB, driving the expression of inflammatory cytokines [33]. Secondly, PM_2.5_ provokes innate immune activation, evidenced by the upregulation of co-stimulatory molecules (e.g., CD86) on antigen-presenting cells and increased secretion of pro-inflammatory cytokines (e.g., IL-6). This may lead to a breakdown of immune regulation and precipitate inflammatory pathology [76]. Thirdly, PM_2.5_ triggers endoplasmic reticulum stress, resulting in the upregulation of pro-apoptotic mediators like CHOP and caspase-12, which promote immune cell death [62]. Finally, PM_2.5_ induces autophagy in macrophages via the oxidative stress-mediated PI3K/AKT/mTOR pathway, contributing to the depletion of these key immune cells [70]. Collectively, these interrelated mechanisms—encompassing oxidative stress, chronic inflammation, organelle stress, and autophagic dysregulation—provide a coherent framework for understanding how PM_2.5_ exposure undermines immune integrity and may precipitate or aggravate autoimmune pathology (Table 1 and Table S3).

#### 5.4.1. PM_2.5_ and Systemic Lupus Erythematosus

Systemic lupus erythematosus (SLE) is a multifactorial autoimmune disorder with a strong female predominance, driven by the interplay of genetic susceptibility and environmental triggers [33]. Recent studies suggest that short-term exposure to PM_2.5_ may exacerbate disease activity and contribute to the progression of systemic lupus erythematosus (SLE) in affected patients [176]. Mechanistically, inhalation of PM_2.5_ can trigger systemic inflammation and oxidative stress, which are central to SLE pathogenesis. Additionally, PM_2.5_ exposure has been shown to disrupt T-helper cell balance (Th1/Th2/Th17), activate B cells and dendritic cells, promote apoptosis, and impair clearance of apoptotic debris, leading to the formation of immune complexes and subsequent renal complications [50]. Consequently, PM_2.5_ constitutes a modifiable environmental risk factor that exacerbates autoimmunity and end-organ damage in SLE, underscoring the critical role of air quality management in clinical and public health strategies (Table 5 and Table S2D).

#### 5.4.2. PM_2.5_ and Rheumatoid Arthritis

Accumulating epidemiological evidence indicates that long-term exposure to PM_2.5_ elevates the risk of developing rheumatoid arthritis (RA). This is supported by large-scale studies, including a prospective cohort which reported a significant positive association and found that combined high genetic susceptibility and high air pollution exposure conferred a substantially increased risk [177]. Mechanistically, PM_2.5_ is thought to promote susceptibility through immunomodulatory pathways, including the activation of the aryl hydrocarbon receptor (AHR), which perturbs the balance between Th17 and regulatory T (Treg) cells, thereby fostering a pro-inflammatory state [33]. Furthermore, PM_2.5_ can trigger systemic inflammation and oxidative stress, and induce epigenetic alterations such as DNA methylation, all of which are implicated in the initiation and progression of RA [33] (Table 5 and Table S2D).

### 5.5. Endocrine System

PM_2.5_ exposure is a significant environmental risk factor for metabolic and endocrine dysfunction, closely linked to type 2 diabetes, insulin resistance, and obesity (Table 6 and Table S2E). The underlying pathogenesis involves a multi-organ cascade, in which oxidative stress plays a central and initiating role. Inhalation of PM_2.5_ triggers reactive oxygen species (ROS) overproduction, which activates interrelated inflammatory (e.g., NF-κB, JNK) and cellular stress (e.g., ER stress, mitochondrial dysfunction) axes [35,51,52]. These pathways impair insulin signaling—particularly via JNK-mediated inhibition of the IRS-1/AKT cascade—leading to systemic insulin resistance. Furthermore, oxidative stress and inflammation disrupt gut microbiota and intestinal barrier function, promoting metabolic endotoxemia that further exacerbates inflammation and metabolic dysfunction [51]. In adipose tissue, PM_2.5_ drives macrophage infiltration and pro-inflammatory polarization, compounding local metabolic disturbances [51]. Thus, through oxidative stress as a common trigger, PM_2.5_ activates interconnected pathways across the lung, gut, and adipose tissue, collectively driving metabolic–endocrine disease (Table 1 and Table S3).

The health effects of PM_2.5_ on the endocrine system are not uniform across the lifespan, with specific developmental and physiological windows conferring heightened susceptibility. Early life—encompassing the prenatal period and early childhood—represents a critical window during which PM_2.5_ exposure can disrupt developmental programming, leading to long-term metabolic consequences such as increased risk of obesity and insulin resistance in offspring [178]. Pregnancy itself constitutes a susceptible period, as PM_2.5_ exposure may exacerbate physiological insulin resistance and contribute to the development of gestational diabetes mellitus [179]. Furthermore, emerging evidence suggests that the menopausal transition represents an understudied yet critical window of vulnerability. Menopause is characterized by declining levels of sex hormones, particularly estrogen, which possesses anti-inflammatory and insulin-sensitizing properties. As these hormones decline, the body’s resilience against environmental contaminants, including PM_2.5_, may diminish, thereby rendering postmenopausal women more susceptible to metabolic dysfunction [180]. Recognizing these vulnerable windows is essential for accurately assessing the public health burden of PM_2.5_ and for developing targeted interventions for high-risk populations.

**Table 6:** Pathogenic mechanisms and evidence levels of PM_2.5_-induced endocrine diseases.

Disease Type	Target/Pathway	Function	Evidence Level
Diabetes	Nrf2/JNK [35]	Oxidative stress and Insulin resistance	▲
	AMPK [181]	Inflammatory response and metabolic disorders	▲
	IL6/STAT3/SOCS3 [182]	Inflammatory response	▲
Obesity (complication—thrombosis)	Proinflammatory cytokines and platelet activation [183]	Thrombosis	★★
	Tlr4/Ikbke [184]	Inflammatory response	▲
	AMPK [181]	Inflammatory response and metabolic disorders	▲
	PPARγ [185]	Adipogenesis	■
	UCP1 [186]	Mitochondrial dysfunction	▲
Thyroid nodule	cross-sectional study [187]	Methylation of DNA, insulin resistance, Inflammatory response and oxidative stress	★★
Hypothyroxinemia	Cohort study [188]	Epidemiology	★★★
Papillary thyroid cancer	Case–control study [189]	Epidemiology	★★
Thyroid dysfunction	Rap1/PI3K/Akt [190]	Thyroid hormone synthesis	▲

Evidence levels: ★★★ = strong human evidence (multiple cohort studies/meta-analyses); ★★ = moderate human evidence (limited human studies or inconsistent results); ▲ = animal studies; ■ = in vitro studies. Combined symbols indicate multiple lines of evidence.

#### 5.5.1. PM_2.5_ and Diabetes

According to the International Diabetes Federation (IDF), an estimated 537 million adults were living with diabetes worldwide in 2021 [191]. Emerging evidence suggests that exposure to PM_2.5_ may be a significant environmental contributor to the incidence and progression of diabetes [192], including gestational diabetes mellitus (GDM), type 1 diabetes (T1D), and type 2 diabetes (T2D) (Table 6). Based on the Global Burden of Disease Study 2021, approximately 20.1% of T2D deaths and 19.4% of T2D-related disability-adjusted life years (DALYs) in China were attributable to air pollution—16.9% and 16.4% from ambient PM_2.5_, and 3.2% and 3.1% from household air pollution, respectively [193]. Epidemiological studies have indicated that the risk of GDM is closely associated with specific chemical components of PM_2.5_, particularly ammonium (NH_4_^+^), organic matter (OM), and nitrate (NO_3_^−^) [179]. In T1D, exposure to PM_2.5_ may exacerbate pancreatic β-cell damage by enhancing pro-inflammatory responses (e.g., IL-1β, TNF-α), which in turn suppresses insulin secretion and ultimately leads to β-cell dysfunction [194]. Regarding the pathogenesis of T2D, experimental studies have demonstrated that PM_2.5_ exposure induces oxidative stress, which in turn activates pathways such as Nrf2/JNK or NF-κB. This activation impairs insulin signaling and disrupts glucose homeostasis, thereby promoting insulin resistance—a central defect in T2D development [35,51]. Additionally, experimental studies demonstrate that PM_2.5_ exacerbates insulin resistance and metabolic dysfunction in T2D by activating the hepatic IL-6/STAT3/SOCS3 signaling pathway [182] (Table 6 and Table S2E). Therefore, by promoting inflammation, oxidative stress, and insulin resistance across different diabetic contexts, PM_2.5_ poses a modifiable environmental risk factor. Converging evidence underscores that reducing ambient PM_2.5_ exposure, particularly in highly polluted regions, is a critical public health strategy for the global prevention and management of diabetes.

#### 5.5.2. PM_2.5_ and Obesity

The global obesity epidemic represents a major public health burden, with growing recognition of the role played by environmental factors. Substantial evidence now positions exposure to PM_2.5_ as a significant environmental risk factor contributing to obesity and its associated metabolic sequelae [195]. For example, exposure to PM_2.5_ was significantly associated with an increased risk of overweight/obesity, with each interquartile range (IQR) increase in PM_2.5_ concentration corresponding to a hazard ratio (HR) of 1.229 (95% CI: 1.137–1.328, *p* < 0.05) [196]. At the cellular level, PM_2.5_ can directly promote adipogenesis and lipid storage by activating the PPARγ pathway in precursor cells, thereby stimulating fat cell development [185]. During critical developmental windows, the study revealed that postnatal exposure to DEP via maternal care promoted weight gain and adiposity in offspring. This metabolic programming was characterized by impaired BAT function, evidenced by tissue whitening and decreased UCP1 expression, suggesting a reduction in energy expenditure [186]. Furthermore, in individuals with established obesity, PM_2.5_ exposure exacerbates cardiovascular risk by specifically promoting a prothrombotic state through platelet activation, an effect significantly mediated by the amplification of obesity-related systemic inflammation [183]. Collectively, these studies reveal that PM_2.5_ contributes to obesity through multiple pathways—directly stimulating fat storage, disrupting developmental metabolism, and amplifying obesity-specific cardiovascular risks (Table 6 and Table S2E).

### 5.6. Digestive System

The digestive system is responsible for the breakdown, absorption, and distribution of nutrients essential for physiological functions. Airborne particulate matter, especially PM_2.5_, can enter the body not only via inhalation but also through ingestion after depositing on food and water, making the digestive system a direct target [197]. The burden of PM_2.5_ on the digestive system is evident at a population level; a 10 μg/m^3^ increase in exposure was associated with a 0.21% increase in hospital admissions for digestive diseases in a two-pollutant model (adjusted *p* < 0.001) [198]. Consistently, Epidemiological and toxicological studies have linked PM_2.5_ exposure to a range of gastrointestinal and hepatic disorders, such as liver injury, fibrosis, hepatitis, gastric and peptic ulcers, irritable bowel syndrome, and malignancies including gastric, hepatic, pancreatic, and esophageal cancers (Table 7 and Table S2F). Notably, heavy metals (e.g., Cd, Cu, Pb, Zn) that are adsorbed onto PM_2.5_—metals characterized by their persistence, toxicity, and bioaccumulative potential—constitute a concern for digestive health via ingestion [199]. At the molecular level, PM_2.5_-induced digestive pathologies are mediated through mechanisms such as oxidative stress, inflammatory activation, mitochondrial dysfunction, autophagy dysregulation, and disruption of gut microbiota homeostasis (Table 1 and Table S3). However, the precise molecular targets and detailed pathways underlying these effects remain to be fully elucidated.

#### 5.6.1. PM_2.5_ and Liver Diseases

A growing body of evidence suggests a significant association between PM_2.5_ exposure and the rising incidence of hepatobiliary disorders. As the body’s primary metabolic hub and the largest exocrine gland, the liver is particularly vulnerable to systemic insults. Upon inhalation, PM_2.5_ can translocate across the alveolar-capillary barrier into the systemic circulation, thereby exerting direct and indirect hepatotoxic effects, including steatosis, inflammatory injury, fibrosis, and even carcinogenesis [206]. The pathogenesis of PM_2.5_-induced liver injury is multifactorial, primarily driven by oxidative stress and inflammatory cascades. Key molecular mechanisms involve activation of the TLR4/MyD88 axis and dysregulation of the Nrf2/SIKE/TBK1/NF-κB signaling network, which collectively exacerbate hepatocellular damage [36,53]. Moreover, PM_2.5_ promotes hepatic fibrogenesis by triggering mitophagy via ROS-induced activation of the PINK1/Parkin pathway, leading to hepatic stellate cell activation and subsequent liver fibrosis [71]. Consistent with this, a murine model study showed that PM_2.5_ impairs liver function by triggering oxidative stress and inflammation via the Nrf2/JNK pathway, resulting in hepatocellular damage and metabolic dysfunction [37]. Chronic hepatic inflammation, which may be driven by exposure to PM_2.5_ and is often indicated by elevated alanine aminotransferase (ALT) levels, is considered a key precursor and a tumor-promoting microenvironment for hepatocellular carcinoma (HCC) [207] (Table 7 and Table S2F). Taken together, evidence from epidemiological, toxicological, and mechanistic studies converges to indicate that PM_2.5_ is not merely a pulmonary pollutant but also a systemic hepatotoxin. Its capacity to induce oxidative stress and persistent inflammation underpins a spectrum of liver pathologies, from metabolic dysfunction to fibrosis and cancer. Acknowledging PM_2.5_ as a modifiable environmental risk factor opens new avenues for the primary prevention of hepatocellular carcinoma and other chronic liver diseases.

#### 5.6.2. PM_2.5_ and Gastrointestinal Diseases

Epidemiological evidence consistently links PM_2.5_ exposure to an increased risk of various gastrointestinal diseases, ranging from chronic malignancies to acute exacerbations (Table 7 and Table S2F). For instance, large-scale cohort studies such as the European ESCAPE project have identified a strong association between long-term exposure to sulfur content within PM_2.5_ and gastric cancer incidence (HR = 1.93), suggesting that specific PM_2.5_ components or correlated pollutants may be key drivers [203]. Experimental research suggests that sulfate particles could enhance the bioavailability of toxic metals, fostering reactive oxygen species generation and DNA damage, while sulfur dioxide derivatives may promote carcinogenic pathways [203]. Beyond carcinogenesis, PM_2.5_ is implicated in broader gastrointestinal pathology through mechanisms involving gut microbiota dysbiosis and direct mucosal injury. Clinical studies indicate that individuals residing in highly polluted areas exhibit an altered gut microbiome, characterized by a decline in beneficial bacteria and an increase in potentially pathogenic taxa [208]. Complementing these chronic alterations, short-term exposure to PM_2.5_ is associated with acute clinical events such as peptic ulcer bleeding, pointing to its role in triggering immediate inflammatory responses or mucosal barrier damage [204]. In summary, PM_2.5_ contributes to digestive tract diseases through multifaceted pathways. These include the chronic, component-specific effects (e.g., sulfur-associated carcinogenesis) and more generalized acute-to-chronic insults mediated by microbial imbalance and direct mucosal injury, underscoring the complex impact of airborne particles on gastrointestinal health.

### 5.7. Genitourinary System

The genitourinary system is essential for excretion and reproduction. The increasing incidence of related disorders has been attributed not only to genetic and lifestyle factors but also to environmental pollutants, with PM_2.5_ as a major concern. PM_2.5_ can traverse biological barriers (e.g., blood–testis, placental, and epithelial barriers), accumulate in reproductive tissues, and induce toxicity. Epidemiological and experimental studies have linked PM_2.5_ exposure to diverse adverse outcomes, including impaired semen quality (e.g., asthenospermia, oligospermia, teratospermia), reduced ovarian reserve, infertility, and a potentially elevated risk of certain reproductive cancers [41,209] (Table 8 and Table S2G).

Studies of its pathogenic mechanisms have been associated with oxidative stress, inflammatory response, cell apoptosis, cell autophagy and DNA methylation (Table 1 and Table S3). Specifically, PM_2.5_ exposure may induce oxidative stress and inflammatory injury, contributing to impaired male reproductive function through activation of the NF-κB/COX-2/PGE_2_ and PI3K/AKT signaling pathways [39,40]. Moreover, PM_2.5_-induced reproductive inflammation involves both pro-inflammatory activation of the NLRP3 inflammasome and compensatory upregulation of the Nrf-2 antioxidant pathway [54,210]. Furthermore, PM_2.5_ can trigger oxidative stress and mitochondrial dysfunction by activating pathways such as PI3K/AKT and TGF-β3/p38 MAPK, which subsequently mediate autophagy and apoptosis, leading to male reproductive impairment [39,66,72,73]. In addition, DNA methylation is closely related to early embryonic development [41]. Collectively, these findings highlight PM_2.5_ as a pervasive reproductive toxicant that acts through interconnected pathological processes.

However, current evidence on the mechanisms of PM_2.5_-induced genitourinary toxicity is largely derived from animal studies. These experimental models have demonstrated that PM_2.5_ can impair reproductive health through oxidative stress, inflammation, apoptosis, and endocrine disruption—pathways that are evolutionarily conserved and likely operative in humans. Importantly, emerging human epidemiological studies have reported outcomes consistent with these mechanistic pathways. For male reproductive health, a nationwide multicenter study of 27,014 Chinese men found that PM_2.5_ exposure was significantly associated with an increased risk of substandard semen quality, with heightened susceptibility in obese individuals [211]. Another study of 1759 men in Wuhan, China, examining key windows of sperm development, reported adverse effects of PM_2.5_ on sperm concentration, count, and motility [212]—parameters that are directly influenced by oxidative stress and germ cell apoptosis in animal models. For female reproductive health, a study analyzing clinical data from 35,989 women in Beijing identified nonlinear associations between short-term exposure to air pollutants (including PM_2.5_) and gynecologic cancer risk [213], aligning with experimental evidence that PM_2.5_ components can induce oxidative DNA damage and endocrine disruption, both of which are implicated in carcinogenesis. Collectively, these findings suggest that the mechanistic pathways identified in animal models are relevant to human reproductive health and provide a foundation for future clinical research. Nevertheless, further large-scale prospective studies with improved exposure assessment are warranted to validate these associations and quantify the true burden of PM_2.5_ on human genitourinary health.

**Table 8:** Pathogenic mechanisms and evidence levels of PM_2.5_-induced genitourinary diseases.

Disease Type	Target/Pathway	Function	Evidence Level
Asthenospermia, oligospermia, deformity	hypothalamic inflammation [214]	Hypothalamic–pituitary–gonadal axis (Suppression)	▲
	UPR/JNK [65]	Apoptosis	▲
	TGF-b3/p38 MAPK [73]	Blood–testis barrier disruption	▲
	ROS, ERS [215]	Oxidative stress, Apoptosis, and DNA damage	▲
	ROS, ATM/P53/CDK2 and Mitochondria apoptosis pathway [216]	Oxidative stress, Apoptosis	▲ + ■
Bladder Cancer	HIF1A/METTL3/IGF2BP3/BIRC5/VEGFA [217]	Angiogenesis and tumor progression	▲ + ■ + ★
Cervical cancer	p53 [218]	Oxidative DNA damage	■
Ovarian cancer	BRCA-1 [219]	Oxidative stress, Chromosomal aberration and Carcinogenesis	★
Breast cancer	prospective cohorts [220]	Oxidative phosphorylation	★★★
	FAK/PI3K/Akt [221]	EMT, cell migration, invasion, metastasis	■
Endometrial cancer	Cross-sectional epidemiological study [222]	Estrogen-like agents interfere with endocrine	★
Prostate cancer	Prospective cohort study [175]	Epidemiology	★★★
Infertility	hypothalamic inflammation [214]	Hypothalamic–pituitary–gonadal axis (Suppression)	▲
	PI3K/Akt/mTOR signaling [223]	Autophagy	▲

Evidence levels: ★★★ = strong human evidence (multiple cohort studies/meta-analyses); ★ = weak human evidence; ▲ = animal studies; ■ = in vitro studies. Combined symbols indicate multiple lines of evidence.

#### 5.7.1. PM_2.5_ and Male Genitourinary Diseases

In males, bladder cancer is one of the most common malignancies of the genitourinary system, with a global male-to-female incidence ratio of approximately 4:1 [224]. In addition to established risk factors such as smoking and occupational exposures, emerging environmental determinants, including PM_2.5_, may serve as potential carcinogens. Epidemiological evidence has demonstrated a positive association between long-term PM_2.5_ exposure and increased bladder cancer mortality [225]. This association is further supported by molecular findings: PM_2.5_ induces METTL3-mediated m^6^A methylation of BIRC5 mRNA, which is subsequently recognized and stabilized by IGF2BP3, leading to BIRC5 overexpression and promoting bladder cancer progression [217].

Furthermore, PM_2.5_ exposure has been associated with abnormal semen parameters (e.g., oligospermia, asthenospermia, teratospermia) and impaired testicular development in offspring (Table 8 and Table S2G). Endocrine disruption represents a key mechanism, primarily involving hypothalamic–pituitary–gonadal (HPG) axis dysfunction. PM_2.5_ induces hypothalamic inflammation, suppressing HPG axis activity and reducing gonadotropin release, thereby downregulating key steroidogenic enzymes (e.g., P450scc, StAR) and affecting testosterone biosynthesis, as well as sperm count, morphology, and concentration [214,226]. Oxidative stress serves as another critical mechanism, with ROS acting as a signaling hub that regulates multiple cellular pathways. For instance, ROS can disrupt blood–testis barrier integrity via the MAPK pathway, inducing germ cell apoptosis [66]; trigger DNA damage and mitochondrial apoptosis through the ATM/p53/CDK2 axis [216]; and synergize with endoplasmic reticulum stress [215], ultimately contributing to male reproductive dysfunction (Table 8 and Table S2G).

However, significant knowledge gaps remain regarding PM_2.5_-induced male reproductive toxicity. For bladder cancer, the precise mechanisms by which PM_2.5_ initiates and promotes tumorigenesis require further investigation. Regarding reproductive function, although multiple pathways have been identified, it remains unclear how these mechanisms interact, whether dose–response relationships exist, and whether findings from animal models can be extrapolated to humans. Future large-scale prospective studies, incorporating refined exposure assessment and integrated in vivo and in vitro experiments, are warranted to comprehensively quantify the true impact of PM_2.5_ on male genitourinary health.

#### 5.7.2. PM_2.5_ and Female Genitourinary Diseases

Breast, cervical, and ovarian cancers are common gynecologic malignancies of the female genitourinary system. Epidemiological studies have demonstrated that PM_2.5_ exposure is associated with adverse reproductive outcomes and an increased risk of gynecologic cancers. For instance, PM_2.5_ can enter systemic circulation and cross biological barriers, leading to impaired ovarian function, reduced embryo quality, and elevated risks of breast, cervical, and ovarian cancers [227,228].

For breast cancer, susceptibility to environmental factors is particularly pronounced in genetically predisposed individuals [229]. Mechanistically, PM_2.5_ activates the AhR-CYP1A1 signaling pathway, leading to reactive oxygen species (ROS) generation, DNA strand breaks, and subsequent PARP-1 activation [230]. These particles also exhibit concentration-dependent estrogenic or anti-estrogenic activities, suggesting their potential as endocrine-disrupting agents [230]. Emerging evidence further indicates that water-soluble components of PM_2.5_ may promote cancer cell metastasis by activating the FAK/PI3K/Akt signaling pathway, thereby accelerating epithelial–mesenchymal transition (EMT) and enhancing cell migration, invasion, and metastatic potential [221].

Regarding cervical cancer, epidemiological evidence supports a link between indoor air pollution from solid fuel combustion (e.g., biomass smoke) and increased cervical cancer mortality, particularly with prolonged exposure [231]. Specific pollutants may synergize with oncogenic HPV infection to accelerate cervical carcinogenesis. For instance, cigarette smoke condensate induces oxidative DNA damage in cervical cells, an effect potentiated in HPV-positive contexts, potentially contributing to genomic instability [218]. Additionally, polycyclic aromatic hydrocarbons (PAHs) such as DMBA upregulate the transcription factor Sp1, which in turn activates Wnt/β-catenin signaling and induces EMT—key processes underlying tumor proliferation and metastasis [232].

For ovarian cancer, an ecological study in Taiwan demonstrated that residential exposure to higher PM_2.5_ levels, particularly traffic-related emissions, was significantly associated with elevated ovarian cancer mortality [233]. Mechanistically, this risk is hypothesized to arise from PM_2.5_-bound PAHs disrupting DNA repair pathways (e.g., BRCA-1) and hormonal balance [219] (Table 8 and Table S2G).

Collectively, these findings indicate that PM_2.5_ may influence the development and progression of female reproductive cancers through multiple interconnected pathways involving oxidative stress, DNA damage, endocrine disruption, and enhanced cellular motility.

### 5.8. Interactions and Overlaps Among Pathogenic Mechanisms

The pathogenic mechanisms of PM_2.5_ described in Section 5.1, Section 5.2, Section 5.3, Section 5.4, Section 5.5, Section 5.6 and Section 5.7 do not operate in isolation; rather, they are interconnected, forming a complex network that collectively drives multisystem toxicity. Elucidating these interactions is essential for a comprehensive understanding of the health effects induced by PM_2.5_.

Oxidative stress serves as the initial step in PM_2.5_ toxicity. Exposure to PM_2.5_ triggers excessive production of reactive oxygen species (ROS), which in turn activate downstream pathways such as inflammation, apoptosis, and autophagy [234]. As an upstream trigger of multiple pathological pathways, ROS play a critical role in PM_2.5_-induced multi-organ damage [234,235]. Therefore, oxidative stress may represent a common pathogenic factor across all affected organ systems.

Endoplasmic reticulum (ER) stress represents another critical shared pathway. PM_2.5_ exposure disrupts ER homeostasis, activating the unfolded protein response (UPR) characterized by upregulation of GRP78, p-IRE1α, and p-p38 [236]. Sustained ER stress leads to apoptosis via CHOP/Caspase12/DR5/Caspase8 activation, contributing to tissue damage in cardiovascular, nervous, and other systems [236]. Importantly, ER stress exhibits significant crosstalk with oxidative stress and inflammation, amplifying cellular injury [236].

Inflammation serves as a central driver of the systemic dissemination of PM_2.5_-induced toxicity. The deposition of PM_2.5_ in the lungs triggers local inflammation, which subsequently spills over into systemic circulation through the release of pro-inflammatory cytokines, including interleukin (IL)-6, IL-8, IL-1β, and tumor necrosis factor-alpha (TNF-α) [235,237]. This systemic inflammatory state then affects distal organs—such as the heart (promoting the initiation and progression of atherosclerosis), the brain (inducing neuroinflammation), and the kidneys—thereby elucidating how respiratory exposure leads to multi-organ pathological damage [235,237].

Apoptosis and autophagy are dynamically balanced. Both processes are regulated by shared signaling pathways, particularly the PI3K/AKT/mTOR axis. PM_2.5_ exposure disrupts this balance by inducing autophagy-mediated cell apoptosis via PI3K/AKT/mTOR signaling [55]. Depending on exposure intensity and cellular context, this balance may tip toward either excessive apoptosis (driving tissue degeneration in COPD and neurodegenerative diseases) or dysregulated autophagy (impairing cellular homeostasis in cardiovascular and reproductive systems) [55,235].

Epigenetic modifications represent another layer of shared mechanism. PM_2.5_ exposure induces DNA methylation, histone modifications, and non-coding RNA alterations that can mediate long-term effects across multiple systems. For example, hypermethylation of the SHANK3 gene leads to synaptic loss and autism-like phenotypes, demonstrating how epigenetic changes can persist after exposure ceases [238].

The gut microbiota has emerged as a critical mediator connecting different organ systems. Studies indicate that inhaled PM_2.5_ can alter gut microbial composition via the gut–lung axis, inducing dysbiosis, which subsequently not only affects digestive health but also exacerbates systemic inflammation and metabolic disturbances [239]. Furthermore, long-term exposure to PM can lead to gut microbiota dysbiosis and alterations in its metabolite profiles, thereby exerting a regulatory feedback effect on pulmonary inflammation and oxidative stress levels [239].

For a conceptual overview of how these pathogenic mechanisms are distributed across different organ systems, reference is made to Supplementary Figure S1.

Collectively, these interactions and overlaps explain why PM_2.5_, despite being primarily inhaled, exerts such broad effects across multiple organ systems [235]. Recognizing this mechanistic network provides a framework for developing multi-target interventions and informs public health strategies aimed at mitigating the full spectrum of PM_2.5_-induced diseases.

## 6. Conclusions

Current research indicates a significant correlation between exposure to PM_2.5_ and the development of diseases affecting the respiratory and cardiovascular systems. The impact of PM_2.5_ exposure on the nervous, immune, endocrine, digestive, and genitourinary systems is increasingly being recognized. Overall, PM_2.5_ poses a multifaceted threat to human health. A significant number of current studies are based on epidemiological surveys with the objective of assessing the correlation between diseases and PM_2.5_. Due to the limitations of traditional epidemiological surveys, it is difficult to explore the types of PM_2.5_-induced diseases in a comprehensive and in-depth manner. The development of multi-omics sequencing technology and the improvement of human disease and gene databases has enabled the establishment of an association network of PM_2.5_–gene–disease from the perspective of molecular epidemiology, which can be used to better explore the types of PM_2.5_-associated diseases at the micro-molecular level. This might represent a significant addition to the traditional epidemiological investigation methods, facilitating a more comprehensive exploration of the relationship between PM_2.5_ and the diseases of various human body systems.

Despite the gradual improvement in global air quality, PM_2.5_ levels in most countries and regions remain above the latest standards set by the World Health Organization, indicating that PM_2.5_ continues to represent a significant threat to the health of the majority of the world’s population. Consequently, the health hazards and pathogenesis of PM_2.5_ have consistently been the focus of extensive investigation. A number of studies have shown that PM_2.5_ has a wide range of effects on the human body through a variety of mechanisms, including the induction of oxidative stress, inflammatory responses, apoptosis, cellular autophagy and genotoxicity, but the complexity of PM_2.5_’s composition and its large geographic and seasonal variations pose a considerable challenge in understanding the potential health risks and the pathogenic mechanisms involved. Future research directions include: strengthening of research on the correlation between PM_2.5_ and diseases in multiple systems of the human body, especially those systems other than the respiratory and cardiovascular systems; taking advantage of the rapid development of disciplines such as molecular biology, multi-omics, and bioinformatics to facilitate multidisciplinary and interdisciplinary research to explore and analyze in depth the pathogenic mechanisms of PM_2.5_ and its integrated effects of multiple systems. In addition, the seasonal and regional variations in PM_2.5_ composition will be taken into account to facilitate a more detailed and multifaceted comprehension of its complexity, diversity and specific hazards. The comprehensive study of PM_2.5_ will enable us to gain a deeper insight into the impact of PM_2.5_ on human health and the underlying pathogenic mechanisms, thereby providing a scientific foundation for the development of effective public health policies and interventions. It should be noted that, due to the substantial geographical and temporal heterogeneity in PM_2.5_ composition and its health effects, quantifying the precise proportion of each disease type within each organ system remains a significant challenge. While the present review does not provide such quantitative estimates, it systematically summarizes the types of diseases associated with PM_2.5_ across multiple systems and the underlying mechanisms, thereby offering a comprehensive framework to guide future research and inform evidence-based health policies.

## Figures and Tables

**Table 1:** Main mechanism of systemic diseases induced by PM_2.5_.

	Systems	Respiration	Cardiovascular	Nerve	Immunity	Endocrine	Digestion	Genitourinary
Mechanisms								
Oxidative stress		PI3K/Akt, NF-κB, JAK-STAT/MAPK, Nrf2-keap1-AREE ^R^ [24]	ROS ^■^ [25] Nrf2 ^▲^ [26] NOX ^■^ [27] AMPK ^▲^ [28] JNK/p53 ^▲^ [29] Nrf2/ARE; NADPH ^R^ [30]	PI3K/Akt/FoxO1 ^▲^ [31] ROS ^R^ [32]	ROS ^R^ [33]; Nrf2 ^▲^ [34]	Nrf2 ^▲^ [35]	Nrf2/SIKE ^▲+■^ [36] Nrf2/JNK ^▲^ [37] NLRP3 ^▲^ [38]	PI3K/Akt ^▲^ [39] NF-κB/COX-2/PGE2 ^▲^ [40] ROS ^R^ [41]
Inflammatory response		NF-κB, JAK-STAT ^R^ [42]; Circ_406961-ILF2-STAT3/JNK ^■^ [43] MAPK/NF-κB/STAT1 ^▲^ [44]	NLRP3 ^■^ [45] AKT/eNOS/NO ^■^ [27] IRAK2/TRAF6/NF-κB ^■^ [46] AMPK ^▲^ [28] JNK/p53 ^▲^ [29] COX-2/PGES/PGE ^■^ [47]	JAK2/STAT3; MAPK; NF-κB ^▲+■^ [48] NLRP3 ^■^ [49] PI3K/Akt/FoxO1 ^▲^ [31]	NF-κB ^▲^ [50] AHR ^R^ [33] Nrf2 ^▲^ [34]	NF-κB ^▲+■^ [51] TLRs/NLRs ^R^ [52]	Nrf2/JNK ^▲^ [37] TLR4/Myd88/NF-κB ^▲^ [53] Nrf2/SIKE ^▲+■^ [36]	NF-κB ^R^ [41] NALP3 ^▲^ [54]
Cell apoptosis		MAPK/NF-κB/STAT1 ^▲^ [44] PI3K/Akt/mTOR ^▲+■^ [55] ATR-CHEK1-TP53 ^■^ [56] NOS2^■^ [57]	Endoplasmic reticulum stress ^R^ [58]; JNK/p53 ^▲^ [29]; IRAK2/TRAF6/NF-κB ^■^ [46]; ROS-Ryr2-Ca2^+ ▲^ [59] COX-2/PGES/PGE ^■^ [47] MAPK; PI3K-AKT^R^ [30]	SAPK; P53 ^R^ [60]; Mitochondria/Endoplasmic reticulum stress ^R^ [61]; PI3K/Akt/FoxO1 ^▲^ [31]	Endoplasmic reticulum stress ^▲^ [62]	Endoplasmic reticulum stress ^R^ [52]		Endoplasmic reticulum stress ^▲^ [63]; NF-κB ^▲^ [64] UPR/JNK ^▲^ [65]; MAPK ^▲^ [66];
Cell autophagy		ERK1/2 and STAT3 ^■^ [67] PI3K/Akt/mTOR ^▲+■^ [55]	Endoplasmic reticulum stress ^R^ [58] Autophagy-related proteins ^R^ [68]	AMPK/mTOR ^■^ [69]	PI3K/AKT/mTOR ^▲^ [70]		PINK1/Parkin/LC3 ^■^ [71]	IRE1/JNK ^▲^ [72] TGF-β3/p38 ^▲^ [73] MAPK ^▲^ [66]
Other factors		Genetic toxicity (DNA and Chromosome damage) ^R^ [3] Ca^2+^ homeostasis imbalance ^▲+■^ [74]	Systemic coagulation abnormalities ^R^ [75] Ca^2+^ homeostasis imbalance ^▲^ [59]	DNA methylation ^R^ [61]	Immune imbalance ^▲^ [76]		Disruption of intestinal microbiota ^▲^ [77]	DNA methylation ^★★^ [78]; Ca^2+^ homeostasis imbalance ^R^ [41]

Evidence levels: ★★ = moderate human evidence (limited human studies or inconsistent results); ▲ = animal studies; ■ = in vitro studies. (R) indicates the mechanism is derived from a review article; evidence level reflects the description of evidence strength in the source review. Abbreviations are defined in Supplementary Table S1. For detailed descriptions of the pathogenic mechanisms, see Tables S2A–G and S3.

**Table 2:** Pathogenic mechanisms and evidence levels of PM_2.5_-induced respiratory diseases.

Disease Type	Target/Pathway	Function	Evidence Level
Lung cancer	ARNT2/PP2A/STAT3/MMP2 [80]	Invasion	▲ + ■
	Wnt3a/β-catenin [81]	Proliferation	▲ + ■
	IL-17a [82]	Proliferation and metastasis	★★★
	lncRNA-loc146880 [83]	Autophagy	■ + ★
	EGFR/PI3K/Akt [84]	invasion and metastasis	■
COPD	Wnt5a/β-catenin [85]	Airway remodeling	▲ + ■
	MAPK and NF-κB [86]	Inflammatory response	▲ + ■
	Wnt5a-JNK [87]	Inflammatory response and fibrosis	▲ + ■ + ★
	CircBbs9-miR-30e-5p-Adar [88]	Inflammatory response	▲
	PI3K/Akt/mTOR [89]	Autophagy, apoptosis	▲ + ■
	NEAT1/PINK1 [90]	Mitophagy	▲ + ■
	METTL16 [91]	Microvascular injury	▲ + ■ + ★
Asthma	NF-κB and MAPK [92]	Inflammatory response and fibrosis	▲ + ■
	JAK-STAT6 [93]	Inflammatory response	▲
	STAT3/RORγt-STAT5/Foxp3 [94]	Immune response	▲
	TLR2/TLR4/MyD88 [95]	Inflammatory response	▲
	Notch signaling pathway [96]	Inflammatory	▲
	TGFβ1/Smad3 [97]	Airway fibrosis	▲
	HMGB1/RAGE [98]	Inflammatory response	▲
Lung injury	ROS-TRPM2-Ca^2+^-NLRP3 [74]	Oxidative stress, inflammatory response and Ca^2+^ homeostasis imbalance	▲ + ■
	AMPK-Beclin1 [99]	Ferroptosis	▲ + ■
	NF-κB [100]	Inflammatory response	▲
	JAK-2/STAT-3 [101]	Inflammatory response and fibrosis	▲ + ■
	IL24/mTOR [102]	Autophagy	▲ + ■
Pulmonary fibrosis	TGFβ-PI3k/Akt, TGFβ1-NOX, TGFβ1-nlrp3 [103]	Inflammatory response and fibrosis	▲
	Akt/mTOR [104]	Oxidative damage and EMT	▲ + ■
Bronchitis	NOS2 [57]	Autophagy	■
	ATR-CHEK1-TP53 [56]	Autophagy	■
COVID-19	NLRP3 [105]	ATP alteration	R
	ACE/ACE2 Pathway [106]	Inflammatory response	▲
Pulmonary eosinophilia	Th2 cell [107]	Immune response	▲
Tuberculosis	Immunity impairment [108]	Immune response	★

Evidence levels: ★★★ = strong human evidence (multiple cohort studies/meta-analyses); ★ = weak human evidence; ▲ = animal studies; ■ = in vitro studies. Combined symbols indicate multiple lines of evidence. (R) indicates the mechanism is derived from a review article.

**Table 5:** Pathogenic mechanisms and evidence levels of PM_2.5_-induced immune diseases.

Disease Type	Target/Pathway	Function	Evidence Level
Systemic lupus erythematosus	NADPH oxidase enzyme [50]	Oxidative stress	▲
	NF-κB [50]	Inflammatory response	
	Th1/Th2/Th17cell [50]	Immune response	
	Cell apoptosis [50]	Apoptosis	
Rheumatoid arthritis	AHR [33]	Inflammatory response and Immune response	R
Viral myocarditis	Th17 cell [170]	Immune response and Inflammatory response	▲
Scleroderma	Epidemiology [171]	Inflammatory response and oxidative stress	★★★
Multiple sclerosis	Oxidative stress, Inflammatory response and DNA methylation alterations [33]	Oxidative stress, Inflammatory response and DNA methylation alterations	R
Sjogren’s syndrome	Epidemiology [171]	Inflammatory response and oxidative stress	★★★
Systemic sclerosis	Epidemiology [171]	Inflammatory response and oxidative stress	★★★
Dermatomyositis	Epidemiology [171]	Inflammatory response and oxidative stress	★★★
Polymyositis	Epidemiology [171]	Inflammatory response and oxidative stress	★★★
Allergic conjunctivitis	Epidemiology [172]	Inflammatory response	★
Allergic rhinitis	ERK-DNMT [173]	Epigenetic regulation and DNA methylation	▲
Polyarteritis nodosa	Epidemiology [171]	Inflammation and oxidative stress	★★★
Membranous nephropathy	IκBα/NF-κB [174]	Inflammatory response	R
	Nrf2/HO-1 and MAPK [174]	Oxidative stress	
	Caspase pathway and NF-κB [174]	Apoptosis	
	DNA damage [174]	DNA damage	
	PKB/mTOR [174]	Autophagy	
Non-Hodgkin’s lymphoma	Prospective cohort study [175]	Epidemiology	★★★

Evidence levels: ★★★ = strong human evidence (multiple cohort studies/meta-analyses); ★ = weak human evidence; ▲ = animal studies. Combined symbols indicate multiple lines of evidence. (R) indicates the mechanism is derived from a review article.

**Table 7:** Pathogenic mechanisms and evidence levels of PM_2.5_-induced digestive diseases.

Disease Type	Target/Pathway	Function	Evidence Level
Nonalcoholic fatty liver disease, liver injury	IRs-1/Akt and CYP2E1/JNK [200]	Insulin Resistance and Oxidative Stress	▲
	Endoplasmic reticulum stress [201]	Inflammatory response	R
	SREBP-1c/FAS [202]	Inflammatory response	▲
	TLR4/myd88 [53]	Inflammatory response	▲
	Nrf2/SIKE [36]	Oxidative stress and Inflammatory response	▲ + ■
Gastric cancer	Epidemiology [203]	Oxidative stress, DNA damage and Genotoxicity	★★★
Peptic ulcer	Time-stratified case-crossover study [204]	Dynamic balance of intestinal microbiota	★★
Irritable Bowel Syndrome	Time-stratified case-crossover study [204]	Dynamic balance of intestinal microbiota	★★
Hepatocellular carcinoma	ROS/Nrf2/keap1 [205]	Autophagy	▲ + ■
Pancreatic cancer	Prospective cohort study [175]	Epidemiology	★★★
Esophageal carcinoma	Prospective cohort study [175]	Epidemiology	★★★
Oral cancer	Prospective cohort study [175]	Epidemiology	★★★
Throat cancer	Prospective cohort study [175]	Epidemiology	★★★

Evidence levels: ★★★ = strong human evidence (multiple cohort studies/meta-analyses); ★★ = moderate human evidence (limited human studies or inconsistent results); ▲ = animal studies; ■ = in vitro studies. Combined symbols indicate multiple lines of evidence. (R) indicates the mechanism is derived from a review article.

## Data Availability

No new data were created or analyzed in this study. Data sharing is not applicable to this article.

## Supplementary Materials

The following supporting information can be downloaded at: https://www.mdpi.com/article/10.3390/toxics14040286/s1, Figure S1: Schematic tree diagram illustrating the conceptual distribution of PM_2.5_-induced pathogenic mechanisms across organ systems; Table S1: Abbreviations of key signaling pathways and molecular targets; Table S2A–G: Detailed pathogenic mechanisms of PM_2.5_-induced multi-system toxicity; Table S3: PM_2.5_-induced detailed pathogenic mechanisms in various systems; Table S4: Summary of literature search and screening by organ system.
