# Supplementary material for: Health Risk and Pathogenesis of PM2.5 in Human Systems
Source: Toxics. 2026 Mar 27;14(4):286. doi: 10.3390/toxics14040286 (PMC13120000; doi:10.3390/toxics14040286)
Supplement: Supplementary file 1 [file toxics-14-00286-s001.zip › Figure S1.pdf]

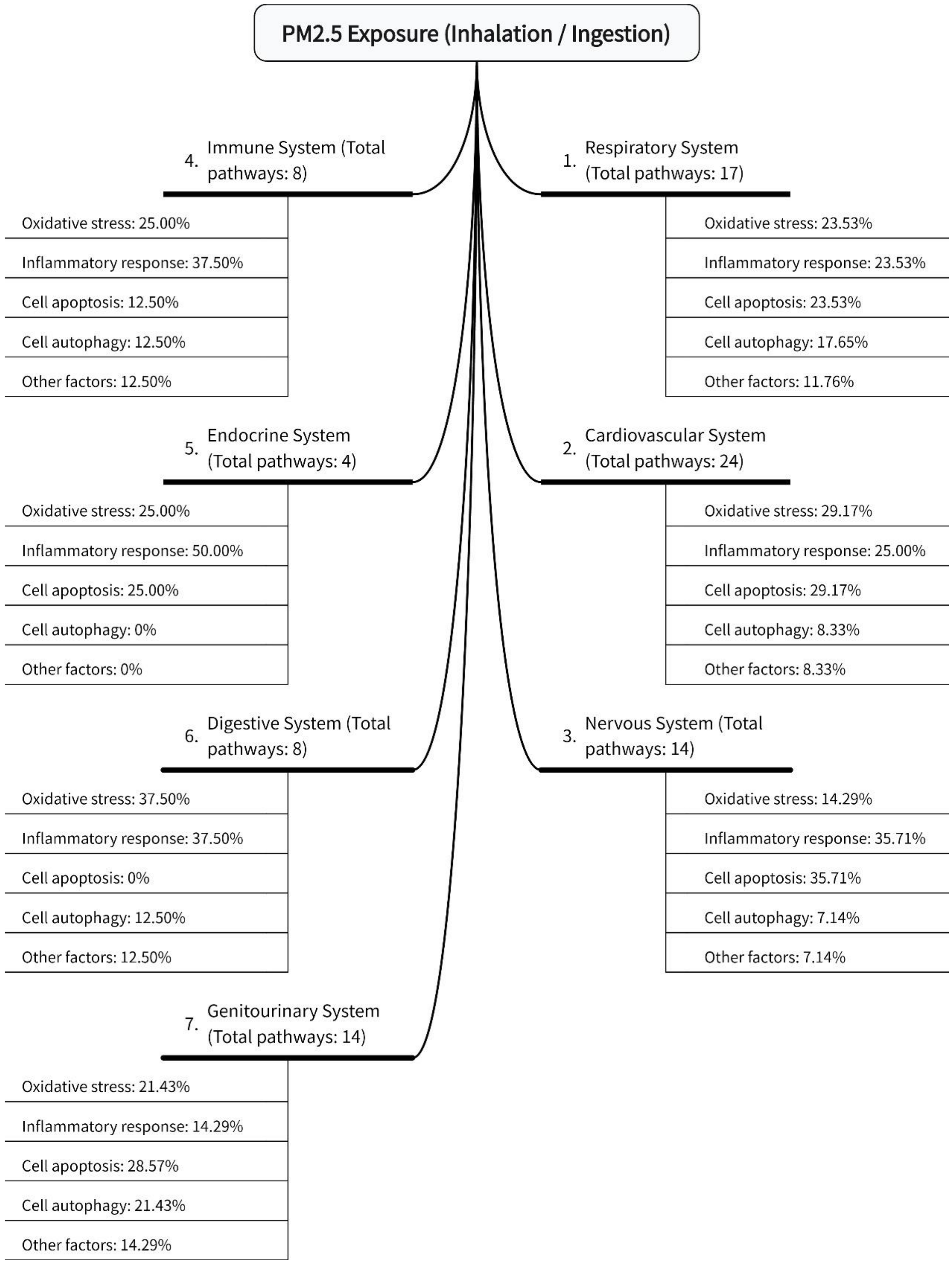

Figure S1. Schematic tree diagram illustrating the conceptual distribution of $PM_{2.5}$-induced pathogenic mechanisms across organ systems.

Note: The diagram originates from $PM_{2.5}$ exposure via inhalation or ingestion. Primary branches represent the seven major organ systems, with the total number of pathway entries for each system (based on Table 1) indicated in parentheses. Secondary branches depict the relative contribution of five key pathogenic mechanisms—oxidative stress, inflammatory response, cell apoptosis, cell autophagy, and other factors—within each system. Percentages were calculated by counting the number of pathway entries for each

mechanism in Table 1 and normalizing to the total pathway count for that system. **This figure is intended as a conceptual illustration only**; the proportions are semi-quantitative estimates and should not be interpreted as precise quantitative measures, as the pathways listed vary in specificity, research depth, and evidence level, and many represent overlapping or interconnected processes rather than discrete, countable units.
