# Supplementary material for: Health Risk and Pathogenesis of PM2.5 in Human Systems
Source: Toxics. 2026 Mar 27;14(4):286. doi: 10.3390/toxics14040286 (PMC13120000; doi:10.3390/toxics14040286)
Supplement: Supplementary file 1 [file toxics-14-00286-s001.zip › Table S1. Abbreviations of key signaling pathways and molecular targets.pdf]

Table S1: Abbreviations of key signaling pathways and molecular targets

Target/Pathway	Full Name
ERK1/2	Extracellular regulated protein kinases 1/2
COX-2/PGES/PGE	Cyclooxygenase-2/ Prostaglandin E synthase/ Prostaglandin E
SAPK	Stress-Activated Protein Kinase
IRE1/JNK	Inositol-requiring enzyme 1/ c-Jun N-terminal kinase pathway
Circ_406961-ILF2-STAT3/JNK	Circular RNA _ 406961- Interleukin Enhancer-Binding Factor 2-Signal Transducer and Activator of Transcription 3/ c-Jun N-terminal Kinase
ARNT2/PP2A/STAT3/MMP2	Aryl Hydrocarbon Receptor Nuclear Translocator 2/Protein Phosphatase 2A/Signal Transducer and Activator of Transcription 3/Matrix Metalloproteinase 2
Wnt3a/β-catenin	Wingless-type MMTV integration site family, member 3A/Beta-catenin
IL-17a	Interleukin-17a
lncRNA-loc146880	Long non-coding RNA – loc146880
EGFR/PI3K/Akt	Epidermal growth factor receptor/phosphatidylinositol 3-kinase/protein kinase B cascade signaling
MAPK	Mitogen-activated protein kinase
NF-KB	Nuclear factor-kappa B (NF-Kb) signaling pathways
Wnt5a-JNK	Wingless-related integration site 5a/c-Jun N-terminal kinase
CircBbs9-miR-30e-5p-Adar	Circular RNA-circBbs9-microRNA-30e-5p
PI3K/Akt/mTOR	Phosphatidylinositol 3-kinase/protein kinase B/mammalian target of rapamycin
NEAT1/ PINK1	Long non-coding ribonucleic acid nuclear enriched abundant transcript 1/ PTEN-induced kinase 1
METTL16	Methyltransferase-like protein 16
JAK-STAT6	Janus kinase 6/signal transducers and activators of transcription 6
STAT3/RORγt-STAT5/Foxp3	Signal Transducer and Activator of Transcription 3/Retinoid-related orphan receptor- Signal Transducer and Activator of Transcription 5/ Forkhead box P3
TLR2/TLR4/MyD88	Toll-like receptor 2/Toll-like receptor 4/Myeloid differentiation factor 88
TGFβ1/Smad3	Transforming growth factor-β1/Smad family member 3
HMGB1/RAGE	High mobility group box 1/Receptor for advanced glycation end products
ROS-TRPM2-Ca^{2+}-NLRP3	Reactive oxygen species-Transient Receptor Potential Melastatin 2-Ca^{2+}-
AMPK-Beclin1	AMP-activated protein kinase/Beclin1
JAK-2/STAT-3	Janus tyrosine protein kinase-2/signal transducer and activator of transcription-3
IL24/mTOR	Interleukin-24/mammalian target of rapamycin
NOS2	Nitric oxide synthase 2

Table S1(Continued)

Target/Pathway	Full Name
eNOS	Endothelial nitric oxide synthase
ATR-CHEK1-TP53	Ataxia Telangiectasia and Rad3-related protein/checkpoint kinase 1/Tumor protein p53
NLRP3	NOD-like receptor family pyrin domain containing 3
ACE/ACE2	Angiotensin-converting enzyme/Angiotensin-converting enzyme 2
Th2 cell	T helper 2 cell
Nox2	NADPH oxidase 2
ET-1	Endothelin-1
Wnt5a/Ror2	Wingless type 5a/Receptor tyrosine kinase-like orphan receptors 2
PVAT	Perivascular adipose tissue
IL-6/gp130/STAT3	Interleukin-6/Glycoprotein 130/Signal Transducer and Activator of Transcription 3
ERK-DNMT	Extracellular regulated protein kinases/DNA methyltransferase
PKB/mTOR	Protein kinase B/mammalian target of rapamycin
AHR	Aryl hydrocarbon receptor
IL6/STAT3/SOCS3	Interleukin-6/Signal Transducer and Activator of Transcription 3/Suppressor of cytokine signaling 3
PPARγ	Peroxisome proliferator activated receptor gamma
UCP1	Uncoupling Protein 1
Rap1/PI3K/Akt	Ras-proximate-1 protein/phosphatidylinositol 3-kinase/protein kinase B
IRs-1/Akt	Insulin receptor substrate-1/protein kinase B
CYP2E1/JNK	Cytochrome P450 2E1/c-jun N-terminal kinase
ERS	Endoplasmic reticulum stress
SREBP-1c/FAS	Sterol regulatory element binding protein-1c/fatty acid synthase
Nrf2/SIKE	Nuclear factor erythroid-derived 2-related factor 2/Suppressor of IKKε
UPR/JNK	Unfolded protein response/c-jun N-terminal kinase
BRCA-1	Breast Cancer 1
PKA/CREB/BDNF	Protein Kinase A/cAMP Response Element-Binding protein/ Brain-Derived Neurotrophic Factor
FoxO1	Forkhead box protein O1
SAPK	Stress Activated Protein Kinase
PINK1/Parkin/LC3	PTEN-induced putative kinase 1/Parkin/microtubule-associated protein 1 light chain 3

This table provides the full names of all abbreviated signaling pathways and molecular targets appearing in the main text and Table 1-2. Abbreviations are listed alphabetically for easy reference.
