# Supplementary material for: Health Risk and Pathogenesis of PM2.5 in Human Systems
Source: Toxics. 2026 Mar 27;14(4):286. doi: 10.3390/toxics14040286 (PMC13120000; doi:10.3390/toxics14040286)
Supplement: Supplementary file 1 [file toxics-14-00286-s001.zip › Table S2. Pathogenic mechanisms and evidence levels of PM2.5-induced diseases by organ system.pdf]

**Table S2A:** Detailed pathogenic mechanisms of $PM_{2.5}$-induced respiratory diseases

| Disease Type | Target/pathway | Pathogenic mechanisms |
|---|---|---|
| Lung cancer | ARNT2/PP2A/STAT3/MMP2[1] | $PM_{2.5}$ promotes lung cancer cell invasion via the ARNT2/PP2A/STAT3/MMP2 pathway |
| | Wnt3a/β-catenin [2] | $PM_{2.5}$ promotes lung cancer progression by inducing exosomal activation of the Wnt3a/β-catenin pathway |
| | IL-17a [3] | Long-term $PM_{2.5}$ exposure promotes lung cancer cell proliferation and metastasis through IL-17a |
| | lncRNA-loc146880 [4] | $PM_{2.5}$ exposure upregulates loc146880 via ROS, mediating autophagy, migration, invasion, and metastasis |
| | EGFR/PI3K/Akt [5] | $PM_{2.5}$ promotes tumor cell migration and invasion via the EGFR-PI3K-AKT pathway |
| COPD | Wnt5a/β-catenin[6] | $PM_{2.5}$ exposure promotes airway remodeling via the Wnt5a/β-Catenin signaling pathway |
| | MAPK and NF-κB[7] | $PM_{2.5}$ induces inflammation via miR-149-5p/TAB2 axis activating MAPK and NF-κB pathways. |
| | Wnt5a-JNK[8] | $PM_{2.5}$ induces lung inflammation and fibrosis via Wnt5a/JNK pathway |
| | CircBbs9-miR-30e-5p-Adar[9] | $PM_{2.5}$ induced inflammation through NLRP3 inflammasome activation regulated by the circBbs9-miR-30e-5p-Adar pathway |
| | PI3K/Akt/mTOR[10] | PI3K/AKT/mTOR pathway regulates autophagy to induce apoptosis of alveolar epithelial cells in COPD induced by $PM_{2.5}$ |
| | NEAT1/PINK1[11] | $PM_{2.5}$ exposure promotes mitophagy via the NEAT1/PINK1 pathway |
| | METTL16[12] | $PM_{2.5}$ induces lung microvascular injury in COPD by METTL16-mediated m6A modification. |
| Asthma | NF-κB and MAPK[13] | $PM_{2.5}$ induces oxidative stress and inflammatory responses via activation of the NF-κB and MAPK signaling pathways |
| | JAK-STAT6[14] | $PM_{2.5}$ exposure aggravates lung inflammation in asthmatic mice through activating the JAK-STAT6 pathway |
| | STAT3/RORγt-STAT5/Foxp3[15] | $PM_{2.5}$ disrupts immune balance in asthmatic mice via the STAT3/RORγt and STAT5/Foxp3 signaling pathways. |
| | TLR2/TLR4/MyD88[16] | $PM_{2.5}$ activates NF-κB via the TLR2/TLR4/MyD88 signaling pathway, thereby aggravating inflammatory responses. |
| | Notch signaling pathway[17] | $PM_{2.5}$ induces Muc5ac secretion via the Notch signaling pathway |
| | TGFβ1/Smad3[18] | $PM_{2.5}$ promotes airway fibrosis via the TGF-β1/Smad3 signaling pathway |
| | HMGB1/RAGE[19] | $PM_{2.5}$ may regulate the HMGB1/RAGE signaling pathway to aggravate airway inflammatory injury in asthma. |
| Lung injury | ROS-TRPM2-$Ca^{2+}$-NLRP3[20] | $PM_{2.5}$ induces acute lung injury via activation of the TRPM2-Ca2+-NLRP3 axis through oxidative stress. |
| | AMPK-Beclin1 [21] | AMPK-Beclin1 signaling pathway positively regulates $PM_{2.5}$-induced ferroptosis |
| | NF-κB[22] | $PM_{2.5}$ induces pulmonary toxicity and inflammatory responses via NF-κB. |
| | JAK-2/STAT-3[23] | $PM_{2.5}$ may mediate inflammatory responses and fibrosis by regulating the JAK-2/STAT-3 signaling pathway, resulting in lung |
| | IL24/mTOR[24] | injury $PM_{2.5}$ may induce pulmonary dysfunction via IL-24-mediated autophagy. |

| | | |
|---|---|---|
| Pulmonary fibrosis | TGFβ-PI3k/Akt, TGFβ1- NOX, TGFβ1-nlrp3[25] | $PM_{2.5}$ induces pulmonary inflammation and fibrosis via the TGFβ1-PI3K/Akt, TGFβ1-NOX, and TGFβ1-NLRP3 signaling pathways. |
| | Akt/mTOR[26] | $PM_{2.5}$ promotes pulmonary fibrosis by inducing oxidative stress and EMT through activation of the AKT/mTOR pathway. |
| Bronchitis | NOS2[27] | $PM_{2.5}$ induces autophagy via NOS2 signaling. |
| | ATR-CHEK1-TP53[28] | $PM_{2.5}$ exposure activates the ATR/CHEK1/CHK1 axis, inducing autophagy and VEGFA production. |
| COVID-19 | NLRP3 [29] | $PM_{2.5}$ may induce alterations in ATR through inflammasome activation. |
| | ACE/ACE2 Pathway[30] | Inflammatory response |
| Pulmonary eosinophilia | Th2 cell[31] | $PM_{2.5}$ exposure acts on the ACE/ACE2 system, thereby affecting COVID-19. |
| Tuberculosis | Immunity impairment[32] | Outdoor $PM_{2.5}$ concentration may be a potential risk factor for the seasonal onset of TB |

**Table S2B:** Detailed pathogenic mechanisms of $PM_{2.5}$-induced cardiovascular diseases

| Disease Type | Target/pathway | Pathogenic mechanisms |
|---|---|---|
| Atherosclerosis | PI3K/Akt/mTOR [33], | $PM_{2.5}$ aggravates atherosclerosis by mediating macrophage apoptosis via the PI3K/Akt/mTOR signaling pathway. |
| | NOX2[34] | NOX2-mediated oxidative stress homeostasis is involved in the process of $PM_{2.5}$-induced atherosclerosis. |
| | Wnt5a/Ror2[35] | Activation of the Wnt5a/Ror2 signaling pathway is involved in the process of $PM_{2.5}$-induced atherosclerosis. |
| | MAPK[36] | The MAPK signaling pathway may play a role in the pathogenesis of PM2.5-induced atherosclerosis. |
| | JAK2/STAT3[37] | $PM_{2.5}$ may induce macrophage lipid accumulation via JAK2/STAT3 inhibition, involving inflammatory responses |
| | IL-6/gp130/STAT3[38] | Inflammatory responses |
| | NLRP3 inflammasome[39] | $PM_{2.5}$ induces endothelial dysfunction by activating the NLRP3 inflammasome. |
| | TLR2/ TLR4/NF-κB and p38/MAPK[40, 41] | Inflammatory responses and Oxidative stress |
| Myocardial infarction | MG53[42] | $PM_{2.5}$ aggravates the severity of myocardial infarction in rats, with the involvement of MG53 protein. |
| | JNK/p53[43] | The JNK/P53 pathway plays a role in $PM_{2.5}$-induced myocardial infarction. |
| | $CD69^+$Treg cells, miR-146a-5p and miR-423-3p[44] | Changes in miR-146a-5p and miR-423-3p are associated with STEMI related to short-term $PM_{2.5}$ exposure. |
| | mitochondrial dysfunction[45] | Mitochondrial dysfunction |
| Arteriosclerotic heart disease | Time series study[46] | Epidemiology |
| Acute coronary syndrome | NO, ET-1 and mitochondria damages[47] | Oxidative stress, vascular tone, vasoconstriction and mitochondria damages |
| Ischemic heart disease | β2AR/PI3K/Akt[48] | $PM_{2.5}$ exposure may cause hypermethylation of myocardial ADRB2, thereby activating the β2AR/PI3K/Akt pathway, leading to $PM_{2.5}$-induced cardiomyocyte apoptosis and cardiac dysfunction. |
| | Oxidative stress and Inflammatory responses[49] | Oxidative stress and Inflammatory responses |
| | PERK/Sestrin2[50] | $PM_{2.5}$ induces cardiomyocyte apoptosis by mediating autophagy via the PERK/Sestrin2 signaling pathway. |
| | NCOA4[51] | $PM_{2.5}$ Induce Endothelial-Mesenchymal Transition and Cardiac Fibrosis via the NCOA4-Mediated ferritinophagy |

| | | |
|---|---|---|
| Atherosclerotic cardiovascular disease | PERK/Sestrin2[50] | $PM_{2.5}$ induces cardiomyocyte apoptosis by mediating autophagy via the PERK/Sestrin2 signaling pathway. |
| Heart failure | Oxidative stress and Inflammatory responses [49]<br>NCOA4[51] | Oxidative stress and Inflammatory responses<br><br>$PM_{2.5}$ Induce Endothelial-Mesenchymal Transition and Cardiac Fibrosis via the NCOA4-Mediated Ferritinophagy |

**Table S2C:** Detailed pathogenic mechanisms of $PM_{2.5}$-induced nervous diseases

| Disease Type | Target/pathway | Pathogenic mechanisms |
|---|---|---|
| Stroke (ischemic and hemorrhagic) | ROS, NLR3P3[52] | Under ischemic conditions, $PM_{2.5}$ triggers NLRP3 inflammasome activation and pyroptosis through ROS-mediated signaling |
| | Akt/eNOS/NO[53] | $PM_{2.5}$ induces endothelial dysfunction and inflammatory injury by upregulating NOX-mediated AKT/eNOS/NO signaling. |
| | Nrf 2/HO-1, NF-κB/TNF-α[54] | PAHs induce oxidative stress and inflammatory responses via the Nrf2/HO-1 and NF-κB/TNF-α pathways, leading to apoptosis |
| | COX-2/PGES/PGE2, | Endothelial damage |
| | ERK/AKT/NF-κB[55] | $PM_{2.5}$ induces a pro-inflammatory phenotype in microglia by regulating TLR4-mediated autophagy and the downstream |
| | PI3K/AKT/NF-κB[55] | PI3K/AKT/NF-κB signaling pathway. |
| Alzheimer's disease | ROS, PI3K/Akt/FoxO1[56] | Oxidative stress |
| | NF-Kb[56], NLRP3[57] | Inflammatory response |
| | AMPK/mTOR[56] | Autophagy |
| | PKA/CREB/BDNF[56] | Neuroprotective effects |
| Neurodevelopmental disorders | Mitochondrial damage[58] | $PM_{2.5}$ exposure during pregnancy induces apoptosis, neuroinflammation, and neurodevelopmental disorders in offspring. |
| | NF-κB, TNF-α, IL-1β[58] | |
| | Caspase family proteins [58] | |
| | SHANK3[59] | The SHANK3 signaling pathway plays a role in autism induced by $PM_{2.5}$ exposure during the early postpartum period. |
| Parkinson's disease | PI3K/Akt/FoxO1[56] | Oxidative stress |
| | NF-κB[56] | Inflammatory response |
| | AMPK/mTOR [56] | Autophagy |
| | PKA/CREB/BDNF [56] | Neuroprotective effects |
| Dementia | PI3K/Akt/FoxO1[56] | Oxidative stress |
| | NF-κB[56] | Inflammatory response |
| | AMPK/mTOR[56] | Autophagy |
| | PKA/CREB/BDNF[56] | Neuroprotective effects |
| Schizophrenia | The striatum[60] | Environmental pollutants affect dopaminergic transmission. |

| | | |
|---|---|---|
| Brain tumor | Epidemiology[56] | Oxidative stress and Inflammatory response |

**Table S2D:** Detailed pathogenic mechanisms of $PM_{2.5}$-induced immune diseases

| Disease Type | Target/pathway | Pathogenic mechanisms |
|---|---|---|
| Systemic lupus erythematosus | NADPH oxidase enzyme[61]<br>NF-κB[61]<br>Th1/Th2/Th17cell[61]<br>Cell apoptosis[61] | Chronic exposure to $PM_{2.5}$ aggravates SLE manifestations |
| Rheumatoid arthritis | AHR[62] | Inflammatory response and Immune response |
| Viral myocarditis | Th17 cell[63] | $PM_{2.5}$ may exacerbate myocardial inflammation via immune suppression and Treg response modulation |
| Scleroderma | Epidemiology[64] | Inflammatory response and oxidative stress |
| Multiple sclerosis | Oxidative stress, Inflammatory response and DNA methylation alterations [62] | Oxidative stress, Inflammatory response and DNA methylation |
| Sjogren's syndrome | Epidemiology[64] | Inflammatory response and oxidative stress |
| Systemic sclerosis | Epidemiology[64] | Inflammatory response and oxidative stress |
| Dermatomyositis | Epidemiology[64] | Inflammatory response and oxidative stress |
| Polymyositis | Epidemiology[64] | Inflammatory response and oxidative stress |
| Allergic conjunctivitis | Epidemiology[65] | Inflammatory response |
| Allergic rhinitis | ERK-DNMT[66] | $PM_{2.5}$ exposure initiates DNA methylation via the ERK-DNMT pathway, aggravating allergic rhinitis. |
| Polyarteritis nodosa | Epidemiology[64] | Inflammation and oxidative stress |

| | | |
|---|---|---|
| Membranous nephropathy | IκBα/NF-κB[67]<br>Nrf2/HO-1 and MAPK[67]<br>Caspase pathway and NF-κB[67]<br>DNA damage[67]<br>PKB/mTOR[67] | Inflammatory response<br>Oxidative stress<br>Apoptosis<br>DNA damage<br>Autophagy |
| Non-Hodgkin's lymphoma | Prospective cohort study[68] | Epidemiology |

**Table S2E:** Detailed pathogenic mechanisms of $PM_{2.5}$-induced endocrine diseases

| Disease Type | Target/pathway | Pathogenic mechanisms |
|---|---|---|
| Diabetes | Nrf2/JNK[69]<br>AMPK[70]<br>IL6/STAT3/SOCS3[71] | $PM_{2.5}$ regulates the Nrf2/JNK signaling pathway to induce hepatic insulin resistance.<br>AMPK may be a potential target in $PM_{2.5}$-induced metabolic diseases<br>Acute $PM_{2.5}$ exposure aggravates type 2 diabetes through the IL-6/STAT3/SOCS3 pathway |
| Obesity (complication - thrombosis) | Proinflammatory cytokines and platelet activation[72]<br>Tlr4/Ikbke[73]<br>AMPK[70]<br>NF-κB[74]<br>PPARγ[75]<br>UCP1[76] | Exposure to fine particulate matter promotes platelet activation and thrombosis via obesity-related inflammation<br>Chronic $PM_{2.5}$ exposure regulates Tlr4/Ikbke to induce hypothalamic inflammation<br>AMPK may be a potential target in $PM_{2.5}$-induced metabolic diseases<br>Oxidative stress<br>$PM_{2.5}$ promotes adipogenesis via activation of PPARγ<br>Mitochondrial dysfunction |
| Thyroid nodule | Epidemiology[77] | Methylation of DNA, insulin resistance, Inflammatory response and oxidative stress |
| Hypothyroxinemia | Cohort study[78] | Epidemiology |
| Papillary thyroid cancer | case-control study [79] | Epidemiology |
| Thyroid dysfunction | Rap1/PI3K/Akt[80] | $PM_{2.5}$ exposure activates GLIS3 via the Rap1/PI3K/AKT pathway, leading to disruption of thyroid hormone homeostasis. |

**Table S2F:** Detailed pathogenic mechanisms of $PM_{2.5}$-induced digestive diseases

| Disease Type | Target/pathway | Pathogenic mechanisms |
|---|---|---|
| Nonalcoholic fatty liver disease, liver injury | IRs-1/Akt and CYP2E1/JNK[81] | Insulin Resistance and Oxidative Stress |
| | Endoplasmic reticulum stress[82] | $PM_{2.5}$ may trigger endoplasmic reticulum stress to induced inflammatory response |
| | SREBP-1c/FAS[83] | $PM_{2.5}$ exposure may promote non-alcoholic steatohepatitis progression by regulating the SREBP-1c/FAS axis. |
| | TLR4/myd88[84] | The TLR4/MyD88 signaling pathway may play a role in $PM_{2.5}$-induced liver injury upon activation. |
| | Nrf2/SIKE[85] | Nrf2/SIKE may be involved in $PM_{2.5}$-induced liver injury. |
| Gastric cancer | Epidemiology[86] | Oxidative stress, DNA damage and Genotoxicity |
| Peptic ulcer | Time-stratified case-crossover study[87] | Dynamic balance of intestinal microbiota |
| Irritable Bowel Syndrome | Time-stratified case-crossover study[87] | Dynamic balance of intestinal microbiota |
| Hepatocellular carcinoma | ROS/Nrf2/keap1[88] | $PM_{2.5}$ induces the development of hepatocellular carcinoma by activating ROS/Nrf2/Keap1-mediated autophagy. |
| Pancreatic cancer | Prospective cohort study[68] | Epidemiology |
| Esophageal carcinoma | Prospective cohort study[68] | Epidemiology |
| Oral cancer | Prospective cohort study[68] | Epidemiology |
| Throat cancer | Prospective cohort study[68] | Epidemiology |

**Table S2G:** Detailed pathogenic mechanisms of $PM_{2.5}$-induced genitourinary diseases

| Disease Type | Target/pathway | Pathogenic mechanisms |
|---|---|---|
| Asthenospermia, oligospermia, deformity | hypothalamic inflammation[89] | $PM_{2.5}$ exposure induces hypothalamic inflammation-mediated HPG axis disruption, leading to spermatogenesis impairment |
| | UPR/JNK[90] | UPR-mediated JNK pathway mediates $PM_{2.5}$-induced testicular cell apoptosis in offspring. |
| | TGF-b3/p38 MAPK pathway[91] | $PM_{2.5}$ disrupts the blood-testis barrier via the TGF-β3/p38 MAPK pathway. |
| | ROS, ERS [92] | $PM_{2.5}$ induces male reproductive toxicity through oxidative and endoplasmic reticulum stress |
| | ROS, ATM/P53/CDK2 and Mitochondria apoptosis pathway[93] | $PM_{2.5}$ induces apoptosis via the ATM/P53/CDK2 and mitochondrial apoptotic pathways |
| Bladder Cancer | HIF1A/METTL3/IGF2BP3/BIRC5/VEGFA[94] | $PM_{2.5}$ exerts epigenetic effects via the HIF1A/METTL3/IGF2BP3/BIRC5/VEGFA network. |
| Cervical cancer | p53[95] | Cigarette smoke condensate (Csc) induces oxidative DNA damage in cervical cells, with repair capacity lower in HPV-positive cells than HPV-negative cells, potentially linked to p53 mutation accumulation |
| Ovarian cancer | BRCA-1[96] | PAH-induced disruption of BRCA-1 expression promotes P53 accumulation, resulting in carcinogenesis |
| Breast cancer | prospective cohorts[97] | $PM_{2.5}$ may contribute to breast carcinogenesis by disrupting oxidative phosphorylation, protein regulation, and cell signaling in adjacent normal breast tissue. |
| | FAK/PI3K/Akt[98] | $PM_{2.5}$ activates the FAK/PI3K/AKT signaling pathway, which promotes epithelial-mesenchymal transition (EMT), thereby enhancing breast cancer cell migration, invasion, and metastatic potential. |
| Endometrial cancer | Cross-sectional epidemiological study [99] | Suspended particles may act as endocrine disruptors and estrogenic factors. |
| Prostate cancer | Prospective cohort study[68] | Epidemiology |

| | | |
|---|---|---|
| Infertility | hypothalamic inflammation[89] | $PM_{2.5}$ exposure induces hypothalamic inflammation-mediated HPG axis disruption, leading to impairment |
| | PI3K/Akt/mTOR signaling[100] | Early exposure to $PM_{2.5}$ induces UPR-mediated autophagy in adult testes via the PI3K/Akt/mTOR pathway. |


1. Chen, Z. Q.; Ji, N. F.; Wang, Z. X.; Wu, C. J.; Sun, Z. X.; Li, Y.; Hu, F.; Wang, Z. B.; Huang, M.; Zhang, M. S., Fine Particulate Matter (PM2.5) Promoted the Invasion of Lung Cancer Cells via an ARNT2/PP2A/STAT3/MMP2 Pathway. *Journal of Biomedical Nanotechnology* **2018,** *14* (12), 2172-2184.
2. Xu, H.; Jiao, X.; Wu, Y.; Li, S.; Cao, L.; Dong, L., Exosomes derived from PM2.5-treated lung cancer cells promote the growth of lung cancer via the Wnt3a/-catenin pathway. *Oncol Rep* **2019,** *41* (2), 1180-1188.
3. Chao, X.; Yi, L.; Lan, L., L, ; Wei, H., Y, ; Wei, D., Long-term PM2.5 exposure increases the risk of non-small cell lung cancer (NSCLC) progression by enhancing interleukin-17a (IL-17a)-regulated proliferation and metastasis. *Aging* **2020,** *12* (12), 11579-11602.
4. Deng, X.; Feng, N.; Zheng, M.; Ye, X.; Lin, H.; Yu, X.; Gan, Z.; Fang, Z.; Zhang, H.; Gao, M.; Zheng, Z.; Yu, H.; Ding, W.; Qian, B., PM2.5 exposure-induced autophagy is mediated by lncRNA loc146880 which also promotes the migration and invasion of lung cancer cells. *Biochim Biophys Acta Gen Subj* **2016,** *1861* (2), 112-125.
5. Lin, H., W, ; Shen, T., J, ; Yang, N., C, ; Wang, M.; Hsieh, W., C, ; Chuang, C., J, ; Lai, C., Y, ; Chang, Y., Y., Luteolin Reduces Aqueous Extract PM2.5-induced Metastatic Activity in H460 Lung Cancer Cells. *Int J Med Sci* **2022,** *19* (10), 1502-1509.
6. Zou, W.; Wang, X.; Sun, R.; Hu, J.; Ye, D.; Bai, G.; Liu, S.; Hong, W.; Guo, M.; Ran, P., PM2.5 Induces Airway Remodeling in Chronic Obstructive Pulmonary Diseases via the Wnt5a/beta-Catenin Pathway. *International journal of chronic obstructive pulmonary disease* **2021,** *16*, 3285-3295.
7. Li, Q.; Li, S.; Xu, C.; Zhao, J.; Hou, L.; Jiang, F.; Zhu, Z.; Wang, Y.; Tian, L., microRNA-149-5p mediates the PM(2.5)-induced inflammatory response by targeting TAB2 via MAPK and NF-κB signaling pathways in vivo and in vitro. *Cell Biol Toxicol* **2023,** *39* (3), 703-717.
8. Zou, W.; Liu, S.; Ye, D.; Bai, G.; Guo, M.; Sun, R.; Ran, P., PM2.5 induces lung inflammation and fibrosis via airway smooth muscle cell expression of the Wnt5a/JNK pathway. *Journal of thoracic disease* **2023,** *15* (11), 6094-6105.
9. Li, M.; Hua, Q.; Shao, Y.; Zeng, H.; Liu, Y.; Diao, Q.; Zhang, H.; Qiu, M.; Zhu, J.; Li, X.; Ling, Y.; Zhang, R.; Jiang, Y., Circular RNA circBbs9 promotes PM(2.5)-induced lung inflammation in mice via NLRP3 inflammasome activation. *Environ Int* **2020,** *143*, 105976.
10. Zhang, F.; Ma, H.; Wang, Z., L, ; Li, W., H, ; Liu, H.; Zhao, Y., X., The PI3K/AKT/mTOR pathway regulates autophagy to induce apoptosis of alveolar epithelial cells in chronic obstructive pulmonary disease caused by PM2.5 particulate matter. *J Int Med Res* **2020,** *48* (7), 1-12.
11. Lin, Q.; Zhang, C. F.; Guo, J. L.; Su, J. L.; Guo, Z. K.; Li, H. Y., Involvement of NEAT1/PINK1-mediated mitophagy in chronic obstructive pulmonary disease induced

by cigarette smoke or PM(2.5). *Ann Transl Med* **2022,** *10* (6), 277.
12. Guo, X. L.; Lin, Y. Y.; Lin, Y. N.; Zhong Y; Yu H; Huang Y; Yang J; Cai Y; Liu F; Li Y; Zhang QQ; J., D., PM2.5 induces pulmonary microvascular injury in COPD via METTL16-mediated m6A modification. *Environ Pollut* **2022,** *303* ((Jun), 119115.1 ~ 119115.12.
13. Zhao, C.; Wang, Y.; Su, Z.; Pu, W.; Niu, M.; Song, S.; Wei, L.; Ding, Y.; Xu, L.; Tian, M.; Wang, H., Respiratory exposure to PM2.5 soluble extract disrupts mucosal barrier function and promotes the development of experimental asthma. *Sci Total Environ* **2020,** *730*, 11.
14. Yang, Y.; Li, X.; An, X.; Zhang, L.; Li, X.; Wang, L.; Zhu, G., Continuous exposure of PM2.5 exacerbates ovalbumin-induced asthma in mouse lung via a JAK-STAT6 signaling pathway. *Adv Clin Exp Med* **2020,** *29* (7), 825-832.
15. Wang, C.; Wang, D.; Zhao, H.; Wang, J.; Liu, N.; Shi, H.; Tian, J.; Wang, X.; Zhang, Z., Traffic-related PM(2.5) and diverse constituents disturb the balance of Th17/Treg cells by STAT3/RORγt-STAT5/Foxp3 signaling pathway in a rat model of asthma. *Int Immunopharmacol* **2021,** *96*, 107788.
16. Wang, L.; Cui, Y.; Liu, H.; Wu, J.; Li, J.; Liu, X., PM2.5 aggravates airway inflammation in asthmatic mice: activating NF-kappa B via MyD88 signaling pathway. *Int J Environ Health Res* **2022**, 1-12.
17. Liu, Y.; Zhou, L.; Wu, H.; Wang, Y.; Dan, z.; Zhang, B., Role of notch signaling pathway in Muc5ac secretion induced by atmospheric PM2.5 in rats. *Ecotoxicol Environ Saf* **2022,** *229*, 113052.
18. Wu, H. Y.; Wang, D.; Shi, H.; Liu, N. N.; Wang, C. H.; Tian, J. Y.; Wang, X.; Zhang, Z. H., PM2.5 and water-soluble components induce airway fibrosis through TGF-beta 1/Smad3 signaling pathway in asthmatic rats. *Molecular Immunology* **2021,** *137*, 1-10.
19. Li, Y.; Chen, L.; Guo, F.; Cao, Y.; Hu, W.; Shi, Y.; Lin, X.; Hou, J.; Li, L.; Ding, X.; Guo, Y., Effects of epigallocatechin-3-gallate on the HMGB1/RAGE pathway in PM2.5-exposed asthmatic rats. *Biochem Biophys Res Commun* **2019,** *513* (4), 898-903.
20. Wang, C.; Meng, X.; Meng, M.; Shi, M.; Sun, W.; Li, X.; Zhang, X.; Liu, R.; Fu, Y.; Song, L., Oxidative stress activates the TRPM2-Ca2+-NLRP3 axis to promote PM2.5-induced lung injury of mice. *Biomedicine & pharmacotherapy = Biomedecine & pharmacotherapie* **2020,** *130* (2020), 110481.
21. Yan, K.; Hou, T.; Zhu, L.; Ci, X.; Peng, L., PM2.5 inhibits system Xc- activity to induce ferroptosis by activating the AMPK-Beclin1 pathway in acute lung injury. *Ecotoxicol Environ Saf* **2022,** *245* (2022), 114083.
22. Yao, H.; Zhao, J.; Zhu, L.; Xie, Y.; Zhao, N.; Yao, R.; Sun, H.; Han, G., Protective effect of the effective part of Andrographis paniculata (Burm.f.) Nees on PM2.5-induced lung injury in rats by modulating the NF-kappa B pathway. *J Ethnopharmacol* **2021,** *280*, 114420.
23. Yue, W.; Chen, X.; He, S.; Li, N.; Zhang, L.; Chen, J., Exposure interval to ambient fine particulate matter (PM2.5) collected in Southwest China induced pulmonary damage through the Janus tyrosine protein kinase-2/signal transducer and activator of transcription-3 signaling pathway both in vivo and in vitro. *J Appl Toxicol* **2021,** *41* (12), 2042-2054.

24. Liu, Y.; He, X.; Liu, J.; Zhang, L.; Xiong, A.; Wang, J.; Liu, S.; Jiang, M.; Luo, L.; Xiong, Y.; Li, G., Transcriptome analysis identifies IL24 as an autophagy modulator in PM2.5 caused lung dysfunction. *Ecotoxicol Environ Saf* **2022,** *244*, 114039.

25. Xu, M.; Wang, X.; Xu, L.; Zhang, H.; Li, C.; Liu, Q.; Chen, Y.; Chung, K.; Adcock, I.; Li, F., Chronic lung inflammation and pulmonary fibrosis after multiple intranasal instillation of PM2.5 in mice. *Environ Toxicol* **2021,** *36* (7), 1434-1446.

26. Zhong, Y., Z, ; Wei, W.; Juan, X.; Guo, H., F., Epigallocatechin Gallate Relieved PM2.5-Induced Lung Fibrosis by Inhibiting Oxidative Damage and Epithelial-Mesenchymal Transition through AKT/mTOR Pathway. *Oxid Med Cell Longev* **2022,** *2022*, 7291774.

27. Zhu, X., M, ; Wang, Q.; Xing, W., W,; Long, M., H, ; Fu, W., L, ; Xia, W., R, ; Jin, C.; Guo, N.; Xu, D., Q, ; Xu, D., G. , PM2.5 induces autophagy-mediated cell death via NOS2 signaling in human bronchial epithelium cells. *Int J Biol Sci* **2018,** *14* (5), 557-564.

28. Xu, X.; Wang, H.; Liu, S.; Xing, C.; Liu, Y.; Aodengqimuge; Zhou, W.; Yuan, X.; Ma, Y.; Hu, M.; Hu, Y.; Zou, S.; Gu, Y.; Peng, S.; Yuan, S.; Li, W.; Ma, Y.; Song, L., TP53-dependent autophagy links the ATR-CHEK1 axis activation to proinflammatory VEGFA production in human bronchial epithelial cells exposed to fine particulate matter (PM2.5). *Autophagy* **2016,** *12* (10), 1832-1848.

29. Zeng, X.; Liu, D.; Wu, W.; Huo, X., PM2.5 exposure inducing ATP alteration links with NLRP3 inflammasome activation. *Environ Sci Pollut Res Int* **2022,** *29* (17), 24445-24456.

30. Botto, L.; Lonati, E.; Russo, S.; Cazzaniga, E.; Bulbarelli, A.; Palestini, P., Effects of PM2.5 Exposure on the ACE/ACE2 Pathway: Possible Implication in COVID-19 Pandemic. *Int J Environ Res Public Health* **2023,** *20* (5).

31. He, M.; Ichinose, T.; Kobayashi, M.; Arashidani, K.; Yoshida, S.; Nishikawa, M.; Takano, H.; Sun, G.; Shibamoto, T., Differences in allergic inflammatory responses between urban PM2.5 and fine particle derived from desert-dust in murine lungs. *Toxicology and applied pharmacology* **2016,** *297*, 41-55.

32. You, S.; Tong, Y., W, ; Neoh, K., G, ; Dai, Y.; Wang, C., H. , On the association between outdoor PM2.5 concentration and the seasonality of tuberculosis for Beijing and Hong Kong. *Environ Pollut* **2016,** *218*, 1170-1179.

33. Wan, Q.; Yang, M.; Liu, Z.; Wu, J., Atmospheric fine particulate matter exposure exacerbates atherosclerosis in apolipoprotein E knockout mice by inhibiting autophagy in macrophages via the PI3K/Akt/mTOR signaling pathway. *Ecotoxicol Environ Saf* **2021,** *208* (2021), 1-10.

34. Liu, J.; Sun, Q.; Sun, M.; Lin, L.; Ren, X.; Li, T.; Xu, Q.; Sun, Z.; Duan, J., Melatonin alleviates PM2.5-triggered macrophage M1 polarization and atherosclerosis via regulating NOX2-mediated oxidative stress homeostasis. *Free Radic Biol Med* **2022,** *181*, 166-179.

35. Wan, Q.; Ding, T.; Xu, Y.; Zheng, C.; Tu, M.; Zhao, T., Urban fine particulate air pollution exposure promotes atherosclerosis in apolipoprotein E-deficient mice by activating perivascular adipose tissue inflammation via the Wnt5a/Ror2 signaling pathway. *Ecotoxicol Environ Saf* **2021,** *227* (2021), 112912.

36. Wang, S.; Wang, F.; Yang, L.; Li, Q.; Huang, Y.; Cheng, Z.; Chu, H.; Song, Y.; Shang, L.; Hao, W.; Wei, X., Effects of coal-fired PM2.5 on the expression levels

of atherosclerosis-related proteins and the phosphorylation level of MAPK in ApoE(-/-) mice. *BMC Pharmacol Toxicol* **2020,** *21* (1), 34.
37. Yang, L.; Song, Z.; Pan, Y.; Zhao, T.; Shi, Y.; Xing, J.; Ju, A.; Zhou, L.; Ye, L., PM2.5 promoted lipid accumulation in macrophage via inhibiting JAK2/STAT3 signaling pathways and aggravating the inflammatory reaction. *Ecotoxicol Environ Saf* **2021,** *226* (2021), 112872.
38. Li, Y., J,; Chen, Y.; Wang, Y., J,; Yao, H., M,; Yang, Q.; Weng, X., G,; Zhu, X., X,; Li, H., Q,; Liu, Y., C., Pathogenesis and Countermeasures of PM2.5 Exposure-induced Instability of Atherosclerotic Plaques. *China Journal of Chinese Materia Medica* **2014,** *39* (15), 5.
39. Hu, T.; Zhu, P.; Liu, Y.; Zhu, H.; Xu, B., PM2.5 induces endothelial dysfunction via activating NLRP3 inflammasome. *Environmental Toxicology* **2021,** *36* (2).
40. Yang, L.; Zhang, Y.; Qi, W.; Zhao, T.; Zhang, L.; Zhou, L.; Ye, L., Adverse effects of PM(2.5) on cardiovascular diseases. *Reviews on environmental health* **2022,** *37* (1), 71-80.
41. Yuan, P., H,; Wu, S., H,; Zhu, M., J,; Zheng, Y., J., The Impact of Fine Particulate Matter (PM2.5) on Myocardial Infarction. *Acta Med Univ Sci Technol Huazhong* **2018,** *47* (6), 4.
42. Zhang, M.; Li, C., R,; Qian, L.; al., e., Effect of fine particulate matter on myocardial infarction and MG53 expression in infracted heart of rats. *Journal of the Third Military Medical University* **2015,** *37* (05), 404-408.
43. Wang, Q.; Gan, X.; Li, F.; Chen, Y.; Fu, W.; Zhu, X.; Xu, D.; Long, M.; Xu, D., PM(2.5) Exposure Induces More Serious Apoptosis of Cardiomyocytes Mediated by Caspase3 through JNK/ P53 Pathway in Hyperlipidemic Rats. *Int J Biol Sci* **2019,** *15* (1), 24-33.
44. Cecconi, A.; Navarrete, G.; Garcia-Guimaraes, M.; Vera, A.; Blanco-Dominguez, R.; Sanz-Garcia, A.; Lozano-Prieto, M.; Lopez-Melgar, B.; Rivero, F.; Martin, P.; Sanchez-Madrid, F.; de la Fuente, H.; Jimenez-Borreguero, L. J.; Alfonso, F., Influence of air pollutants on circulating inflammatory cells and microRNA expression in acute myocardial infarction. *Sci Rep* **2022,** *12* (1), 5350.
45. Sivakumar, B.; Kurian, G. A., Inhalation of PM2.5 from diesel exhaust promote impairment of mitochondrial bioenergetics and dysregulate mitochondrial quality in rat heart: implications in isoproterenol-induced myocardial infarction model. *Inhal Toxicol* **2022,** *34* (5-6), 107-119.
46. Zhou, X.; Wang, C.; Chen, P.; Chen, Y.; Yin, L.; Du, W.; Pu, Y., Time series analysis of short-term effects of particulate matter pollution on the circulatory system disease mortality risk in Lishui District, China. *Environ Sci Pollut Res Int* **2022,** *29* (12), 17520-17529.
47. Meng, X.; Zhang, Y.; Yang, K., Q, ; Yang, Y., K, ; Zhou, X., L. , Potential Harmful Effects of PM2.5 on Occurrence and Progression of Acute Coronary Syndrome: Epidemiology, Mechanisms, and Prevention Measures. *Int J Environ Res Public Health* **2016,** *13* (8), 748.
48. Yang, X.; Zhao, T.; Feng, L.; Shi, Y.; Jiang, J.; Liang, S.; Sun, B.; Xu, Q.; Duan, J.; Sun, Z., PM(2.5)-induced ADRB2 hypermethylation contributed to cardiac dysfunction through cardiomyocytes apoptosis via PI3K/Akt pathway. *Environ Int* **2019,** *127*, 601-614.
49. Zhao, T.; Qi, W.; Yang, P.; Yang, L.; Shi, Y.; Zhou, L.; Ye, L., Mechanisms of cardiovascular toxicity induced by PM(2.5): a review. *Environ Sci Pollut Res Int* **2021,** *28*

(46), 65033-65051.
50. Tian, J.; Niu, Z.; Yang, H.; Wang, C.; Guan, L.; Zhao, L.; Shi, D.; Zhang, Z., PERK/Sestrin2 Signaling Pathway Mediated Autophagy Regulates Human Cardiomyocytes Apoptosis Induced by Traffic-Related PM2.5 and Diverse Constituents. *International Journal of Molecular Sciences* **2025,** *26* (8), 3784.
51. Sun, Q.; Wang, M.; Liu, L.; Ding, R.; Yan, K.; Liu, S.; Ren, X.; Xu, Q.; Sun, Z.; Liu, Q.; Yang, Y.; Duan, J., PM(2) (.5) Induce Endothelial-Mesenchymal Transition and Cardiac Fibrosis via the NCOA4-Mediated Ferritinophagy. *Advanced science (Weinheim, Baden-Wurttemberg, Germany)* **2025,** *12* (45), e07536.
52. Gao, L.; Qin, J. X.; Shi, J. Q.; Jiang, T.; Wang, F.; Xie, C.; Gao, Q.; Zhi, N.; Dong, Q.; Guan, Y. T., Fine particulate matter exposure aggravates ischemic injury via NLRP3 inflammasome activation and pyroptosis. *Cns Neuroscience & Therapeutics* **2022,** *28* (7), 1045-1058.
53. Zou, L.; Xiong, L.; Wu, T.; Wei, T.; Liu, N.; Bai, C.; Huang, X.; Hu, Y.; Xue, Y.; Zhang, T.; Tang, M., NADPH oxidases regulate endothelial inflammatory injury induced by PM(2.5) via AKT/eNOS/NO axis. *J Appl Toxicol* **2022,** *42* (5), 738-749.
54. He, J.; Pang, Q.; Huang, C.; Xie, J.; Hu, J.; Wang, L.; Wang, C.; Meng, L.; Fan, R., Environmental dose of 16 priority-controlled PAHs mixture induce damages of vascular endothelial cells involved in oxidative stress and inflammation. *Toxicol In Vitro* **2022,** *79*, 105296.
55. Chen, Z.; Liu, P.; Xia, X.; Wang, L.; Li, X., The underlying mechanism of PM2.5-induced ischemic stroke. *Environ Pollut* **2022,** *310*, 119827.
56. Li, W.; Lin, G.; Xiao, Z.; Zhang, Y.; Li, B.; Zhou, Y.; Ma, Y.; Chai, E., A review of respirable fine particulate matter (PM2.5)-induced brain damage. *Front Mol Neurosci* **2022,** *15*, 1-34.
57. Shi, J. Q.; Wang, B. R.; Jiang, T.; Gao, L.; Zhang, Y. D.; Xu, J., NLRP3 Inflammasome: A Potential Therapeutic Target in Fine Particulate Matter-Induced Neuroinflammation in Alzheimer's Disease. *Journal of Alzheimers Disease* **2020,** *77* (3), 923-934.
58. Zheng, X.; Wang, X.; Wang, T.; Zhang, H.; Wu, H.; Zhang, C.; Yu, L.; Guan, Y., Gestational Exposure to Particulate Matter 2.5 (PM2.5) Leads to Spatial Memory Dysfunction and Neurodevelopmental Impairment in Hippocampus of Mice Offspring. *Front Neurosci* **2019,** *12*, 1-18.
59. Li, K.; Liang, X. T.; Xie, X. Q.; Tian, L.; Yan, J.; Lin, B. C.; Liu, H. L.; Lai, W. Q.; Liu, X. H.; Xi, Z. G., Role of SHANK3 in concentrated ambient PM2. 5 exposure induced autism-like phenotype. *Heliyon* **2023,** *9* (3).
60. Andrade-Oliva MD; Escamilla-Sánchez J; Debray-García Y; Morales-Rubio RA; González-Pantoja R; Uribe-Ramírez M; Amador-Muñoz O; Díaz-Godoy RV; De Vizcaya-Ruiz A; JA., A.-M., In vitro exposure to ambient fine and ultrafine particles alters dopamine uptake and release, and D-2 receptor affinity and signaling. *Environ Toxicol Pharmacol* **2020,** *80*, 103484-103495.
61. Yariwake VY; Torres JI; Dos Santos ARP; Freitas SCF; De Angelis K; Farhat SCL; Câmara NOS; MM., V., Chronic exposure to PM2.5 aggravates SLE manifestations in lupus-prone mice. *Part Fibre Toxicol* **2021,** *18* (1), 15.
62. Zhao, C., N, ; Xu, Z.; Wu, G., C, ; Mao, Y., M, ; Liu, L., N, ; Qian, W.; Dan, Y., L, ; Tao, S., S, ; Zhang, Q.; Sam, N., B, ; Fan, Y., G, ; Zou, Y., F, ; Ye, D., Q, ;

Pan, H., F. , Emerging role of air pollution in autoimmune diseases. *Autoimmun Rev* **2019,** *18* (6), 607-614.
63. Xie, Y.; Gong, C.; Bo, L.; Jiang, S.; Kan, H.; Song, W.; Zhao, J.; Li, Y., Treg responses are associated with PM2.5-induced exacerbation of viral myocarditis. *Inhal Toxicol* **2015,** *27* (6), 281-286.
64. Zhao, N.; Smargiassi A; Jean S; Gamache P; Laouan-Sidi EA; Chen, H.; Goldberg MS; S., B., Long-term exposure to fine particulate matter and ozone and the onset of systemic autoimmune rheumatic diseases: an open cohort study in Quebec, Canada. *Arthritis research & therapy* **2022,** *24* (1), 151.
65. Mimura T; Ichinose T; Yamagami S; Fujishima H; Kamei Y; Goto M; Takada S; M., M., Airborne particulate matter (PM2.5) and the prevalence of allergic conjunctivitis in Japan. *Sci Total Environ* **2014,** *487*, 493-499.
66. Li, Y.; Zhou, J.; Rui, X.; Zhou, L.; Mo, X., PM2.5 exposure exacerbates allergic rhinitis in mice by increasing DNA methylation in the IFN- γ gene promoter in CD4+T cells via the ERK-DNMT pathway. *Toxicol Lett* **2019,** *301*, 98-107.
67. Xu, W.; Wang, S.; Jiang, L.; Sun, X.; Wang, N.; Liu, X.; Yao, X.; Qiu, T.; Zhang, C.; Li, J.; Deng, H.; Yang, G., The influence of PM2.5 exposure on kidney diseases. *Hum Exp Toxicol* **2022,** *41*, 1-9.
68. Shin M; Kim OJ; Yang S; Choe SA; SY., K., Different Mortality Risks of Long-Term Exposure to Particulate Matter across Different Cancer Sites. *Int J Environ Res Public Health* **2022,** *19* (6), 3180.
69. Xu J; Zhang W; Lu Z; Zhang F; W., D., Airborne PM2.5-Induced Hepatic Insulin Resistance by Nrf2/JNK-Mediated Signaling Pathway. *Int J Environ Res Public Health* **2017,** *14* (7), 1-15.
70. Pan, K.; Jiang, S.; Du, X.; Zeng, X.; Zhang, J.; Song, L.; Zhou, J.; Kan, H.; Sun, Q.; Xie, Y.; Zhao, J., AMPK activation attenuates inflammatory response to reduce ambient PM2.5-induced metabolic disorders in healthy and diabetic mice. *Ecotoxicol Environ Saf* **2019,** *179* (Sep.), 290-300.
71. Long, M. H.; Zhang, C.; Xu, D. Q.; Fu, W. L.; Gan, X. D.; Li, F.; Wang, Q.; Xia, W.; Xu, D. G., PM(2.5) aggravates diabetes via the systemically activated IL-6-mediated STAT3/SOCS3 pathway in rats' liver. *Environ Pollut* **2020,** *256*, 113342.
72. Hu, D.; Jia, X.; Cui, L.; Liu, J.; Chen, J.; Wang, Y.; Niu, W.; Xu, J.; Miller, M.; Loh, M.; Deng, F.; Guo, X., Exposure to fine particulate matter promotes platelet activation and thrombosis via obesity-related inflammation. *J Hazard Mater* **2021,** *413* (Jul.5), 125341.1 ~ 125341.9.
73. Campolim, C., M,; Weissmann, L.; Ferreira CKO; Zordão OP; Dornellas APS; de Castro G; Zanotto TM; Boico VF; Quaresma PGF; Lima RPA; Donato J Jr; Veras MM; Saldiva PHN; Kim YB; PO., P., Short-term exposure to air pollution (PM2.5) induces hypothalamic inflammation, and long-term leads to leptin resistance and obesity via Tlr4/Ikbke in mice. *Sci Rep* **2020,** *10* (1), 10160.
74. Wang, N.; Ma, Y.; Liu, Z.; Liu, L.; Yang, K.; Wei, Y.; Liu, Y.; Chen, X.; Sun, X.; Wen, D., Hydroxytyrosol prevents PM2.5-induced adiposity and insulin resistance by restraining oxidative stress related NF-kappa B pathway and modulation of gut microbiota in a murine model. *Free Radic Biol Med* **2019,** *141*, 393-407.

75. Cao, Y. Q.; Chen, Y. Y.; Miao, K.; Zhang, S. Y.; Deng, F. C.; Zhu, M.; Wang, C.; Gu, W.; Huang, Y. X.; Shao, Z. J.; Dong, X. Y.; Gong, Y. F.; Peng, H.; Yang, H.; Wan, Y.; Jia, X. D.; Tang, S., PPARγ As a Potential Target for Adipogenesis Induced by Fine Particulate Matter in 3T3-L1 Preadipocytes. *Environmental Science & Technology* **2023,** *57* (20), 7684-7697.
76. Chen, M.; Liang, S.; Zhou, H.; Xu, Y.; Qin, X.; Hu, Z.; Wang, X.; Qiu, L.; Wang, W.; Zhang, Y.; Ying, Z., Prenatal and postnatal mothering by diesel exhaust PM2.5-exposed dams differentially program mouse energy metabolism. *Part Fibre Toxicol* **2017,** *14* (1), 3.
77. Zhang, Y.; Wang, K.; Qin, W.; Jin, C.; Song, Y.; Jia, P.; Wang, S.; Song, Y.; Ning, Y.; Li, L., Six Air Pollutants Associated With Increased Risk of Thyroid Nodules: A Study of 4.9 Million Chinese Adults. *Front Endocrinol (Lausanne)* **2021,** *12*, 753607.
78. Ghassabian A; Pierotti L; Basterrechea M; Chatzi L; Estarlich M; Fernández-Somoano A; Fleisch AF; Gold DR; Julvez J; Karakosta P; Lertxundi A; Lopez-Espinosa MJ; Mulder TA; Korevaar TIM; Oken E; Peeters RP; Rifas-Shiman S; Stephanou E; Tardón A; Tiemeier H; Vrijheid M; Vrijkotte TGM; Sunyer J; M., G., Association of Exposure to Ambient Air Pollution With Thyroid Function During Pregnancy. *JAMA Netw Open* **2019,** *2* (10), e1912902.
79. Crepeau, P.; Zhang, Z.; Udyavar, R.; Morris-Wiseman, L.; Biswal, S.; Ramanathan, M., Jr.; Mathur, A., Socioeconomic disparity in the association between fine particulate matter exposure and papillary thyroid cancer. *Environ Health* **2023,** *22* (1), 20.
80. Tang, S.; Li, D.; Ding, H.; Jiang, M.; Zhao, Y.; Yu, D.; Zhang, R.; Chen, W.; Chen, R.; Zheng, Y.; Piao, J., GLIS3 mediated by the Rap1/PI3K/AKT signal pathway facilitates real-ambient PM2.5 exposure disturbed thyroid hormone homeostasis regulation. *Ecotoxicol Environ Saf* **2022,** *232* (2022), 113248.
81. Jian, T.; Ding, X.; Wu, Y.; Ren, B.; Li, W.; Lv, H.; Chen, J., Hepatoprotective Effect of Loquat Leaf Flavonoids in PM2.5-Induced Non-Alcoholic Fatty Liver Disease via Regulation of IRs-1/Akt and CYP2E1/JNK Pathways. *Int J Mol Sci* **2018,** *19* (10), 3005.
82. Chen, J.; Wu, L.; Yang, G.; Zhang, C.; Liu, X.; Sun, X.; Chen, X.; Wang, N., The influence of PM(2.5 )exposure on non-alcoholic fatty liver disease. *Life Sci* **2021,** *270* (7), 119135.
83. Ding, S.; Yuan, C.; Si, B.; Wang, M.; Da, S.; Bai, L.; Wu, W., Combined effects of ambient particulate matter exposure and a high-fat diet on oxidative stress and steatohepatitis in mice. *Plos One* **2019,** *14* (3), e0214680.
84. Ya, P.; Xu, H.; Ma, Y.; Fang, M.; Yan, X.; Zhou, J.; Li, F., Liver injury induced in Balb/c mice by PM(2.5) exposure and its alleviation by compound essential oils. *Biomedicine & pharmacotherapy = Biomedecine & pharmacotherapie* **2018,** *105*, 590-598.
85. Ge, C. X.; Tan, J.; Zhong, S. Y.; Lai, L. L.; Chen, G.; Zhao, J. J.; Yi, C.; Wang, L. Y.; Zhou, L. W.; Tang, T. T.; Yang, Q. F.; Lou, D. S.; Li, Q.; Wu, Y. K.; Hu, L. F.; Kuang, G.; Liu, X.; Wang, B. C.; Xu, M. X., Nrf2 mitigates prolonged PM2.5 exposure-triggered liver inflammation by positively regulating SIKE activity: Protection by Juglanin. *Redox Biology* **2020,** *36*, 101645.
86. Weinmayr G; Pedersen M; Stafoggia M; Andersen ZJ; Galassi C; Munkenast J; Jaensch A; Oftedal B; Krog NH; Aamodt G; Pyko A; Pershagen G; Korek

M; De Faire U; Pedersen NL; Östenson CG; Rizzuto D; Sørensen M; Tjønneland A; Bueno-de-Mesquita B; Vermeulen R; Eeftens M; Concin H; Lang A; Wang M; Tsai MY; Ricceri F; Sacerdote C; Ranzi A; Cesaroni G; Forastiere F; de Hoogh K; Beelen R; Vineis P; Kooter I; Sokhi R; Brunekreef B; Hoek G; Raaschou-Nielsen O; G., N., Particulate matter air pollution components and incidence of cancers of the stomach and the upper aerodigestive tract in the European Study of Cohorts of Air Pollution Effects (ESCAPE) *Environ Int* **2018,** *120*, 163-171.

87. Wu, M. Y.; Tang, M. L.; Yu, Z. B.; al., e., Association between short-term exposure to air pollution and peptic ulcer bleeding: A case-crossover study in China. *ATMOS ENVIRON* **2021,** *256* (Jul.), 118438.1 ~ 118438.8

88. Li, J.; Jiang, H.; Zhu, Y.; Ma, Z.; Li, B.; Dong, J.; Xiao, C.; Hu, A., Fine particulate matter (PM(2.5)) induces the stem cell-like properties of hepatocellular carcinoma by activating ROS/Nrf2/Keap1-mediated autophagy. *Ecotoxicol Environ Saf* **2024,** *272*, 116052.

89. Qiu, L.; Chen, M.; Wang, X.; Qin, X.; Chen, S.; Qian, Y.; Liu, Z.; Cao, Q.; Ying, Z., Exposure to Concentrated Ambient PM2.5 Compromises Spermatogenesis in a Mouse Model: Role of Suppression of Hypothalamus-Pituitary-Gonads Axis. *Toxicol Sci* **2018,** *162* (1), 318-326.

90. Ren, L.; Jiang, J.; Huang, J.; Zang, Y.; Huang, Q.; Zhang, L.; Wei, J.; Lu, H.; Wu, S.; Zhou, X., Maternal exposure to PM2.5 induces the testicular cell apoptosis in offspring triggered by the UPR-mediated JNK pathway. *Toxicol Res (Camb)* **2022,** *11* (1), 226-234.

91. Liu, J.; Ren, L.; Wei, J.; Zhang, J.; Zhu, Y.; Li, X.; Jing, L.; Duan, J.; Zhou, X.; Sun, Z., Fine particle matter disrupts the blood-testis barrier by activating TGF-beta 3/p38 MAPK pathway and decreasing testosterone secretion in rat. *Environ Toxicol* **2018,** *33* (7), 711-719.

92. Liu, H. L.; Ding, S. S.; Nie, H. P.; Shi, Y.; Lai, W. Q.; Liu, X.; Li, K.; Tian, L.; Xi, Z. G.; Lin, B. C., PM2.5 exposure at different concentrations and modes induces reproductive toxicity in male rats mediated by oxidative and endoplasmic reticulum stress. *Ecotoxicology and Environmental Safety* **2022,** *244*, 114042.

93. Liu, J.; Zhang, J.; Ren, L.; Wei, J.; Zhu, Y.; Duan, J.; Jing, L.; Sun, Z.; Zhou, X., Fine particulate matters induce apoptosis via the ATM/P53/CDK2 and mitochondria apoptosis pathway triggered by oxidative stress in rat and GC-2spd cell. *Ecotoxicol Environ Saf* **2019,** *180* (Sep.), 280-287.

94. Liu, H.; Gu, J.; Huang, Z.; Han, Z.; Xin, J.; Yuan, L.; Du, M.; Chu, H.; Wang, M.; Zhang, Z., Fine particulate matter induces METTL3-mediated m(6)A modification of BIRC5 mRNA in bladder cancer. *J Hazard Mater* **2022,** *437*, 129310.

95. Moktar A; Singh R; Vadhanam MV; Ravoori S; Lillard JW; Gairola CG; RC., G., Cigarette smoke condensate-induced oxidative DNA damage and its removal in human cervical cancer cells. *International journal of oncology* **2011,** *39* (4), 941-947.

96. Wang, H.; Zhou, Y.; Yang, Y., J, ; Li, Y., B, ; Feng, Y., An ecological study on the urban-rural differences in the increased risk of ovarian cancer caused by PM2.5. *China Environmental Science* **2019,** *39* (1), 6.

97. DuPre, N. C.; Heng, Y. J. J.; Raby, B. A.; al., e., Involvement of fine particulate matter exposure with gene expression pathways in breast tumor and adjacent-normal breast tissue. *Environ Res* **2020,** *186*, 109535.

98. Cheng, C., W, ; Sheu, G., T, ; Chou, J., S, ; Wang, P., H, ; Cheng, Y., C,; Lai, C., Y., Fine particulate matter PM2.5 generated by building demolition increases the malignancy of breast cancer MDA-MB-231 cells. *Chemosphere* **2021,** *265* (Feb), 10.

99. Iwai, K.; Mizuno, S.; Miyasaka, Y.; T., M., Correlation between suspended particles in the environmental air and causes of disease among inhabitants: Cross-sectional studies using the vital statistics and air pollution data in Japan. *Environ Res* **2005,** *99* (1), 106-117.

100. Ren, L.; Huang, J.; Wei, J.; Zang, Y.; Zhao, Y.; Wu, S.; Zhao, X.; Zhou, X.; Sun, Z.; Lu, H., Maternal exposure to fine particle matters cause autophagy via UPR-mediated PI3K-mTOR pathway in testicular tissue of adult male mice in offspring. *Ecotoxicol Environ Saf* **2020,** *189*, 109943.1 ~ 109943.9.
