# Supplementary material for: Health Risk and Pathogenesis of PM2.5 in Human Systems
Source: Toxics. 2026 Mar 27;14(4):286. doi: 10.3390/toxics14040286 (PMC13120000; doi:10.3390/toxics14040286)
Supplement: Supplementary file 1 [file toxics-14-00286-s001.zip › Table S3.pdf]

Table S3: $PM_{2.5}$-induced detailed pathogenic mechanisms in various systems

Systems	Target/Pathway	Pathogenic mechanisms
Respiration	MAPK/NF-κB/STAT1▲[1]	The MAPK/NF-κB/STAT1 pathway mediates COF-$PM_{2.5}$-induced inflammation, apoptosis, and cellular damage in A549 cells.
	PI3K/Akt/mTOR▲+■[2]	$PM_{2.5}$ induces autophagy-mediated cell apoptosis via PI3K/AKT/mTOR signaling pathway
	ATR-CHEK1-TP53 ■[3]	$PM_{2.5}$ exposure triggers autophagy and VEGFA production via the ATR-CHEK1/CHK1 axis.
	Circ_406961-ILF2-STAT3/JNK■[4]	$PM_{2.5}$ induces inflammation through the circRNA 406961/ILF2-mediated STAT3/JNK pathway
	NOS2■[5]	$PM_{2.5}$ induces autophagy-mediated cell death through the NOS2 pathway.
	ERK1/2 and STAT3 ■[6]	ERK1/2 and STAT3 are involved in $PM_{2.5}$-induced apoptosis in A549 cells.
	Ca^{2+} homeostasis imbalance ▲+■[7]	$PM_{2.5}$ promotes lung injury by regulating the TRPM2-Ca^{2+}-NLRP3 axis via oxidative stress.
Cardiovascular	JNK/p53▲[8];	JNK/P53 is critical in $PM_{2.5}$-induced inflammation, oxidative stress, and apoptosis in circulatory system diseases.
	IRAK2/TRAF6/NF-κB ■[9]	$PM_{2.5}$ could trigger myocardial toxicity via miR-205 negative regulating the IRAK2/TRAF6/NF-κB signaling pathway.
	ROS-Ryr2-Ca2+ ▲[10]	$PM_{2.5}$ induces cardiac injury via Ryr2-Ca^{2+}-mediated apoptosis through oxidative stress.
	COX-2/PGES/PGE ■[11]	$PM_{2.5}$ may promote apoptosis and inflammation by activating the COX-2/PGES/PGE2 axis.
	Nrf2▲[12]	Nrf2 is involved in PM-induced cardiovascular injury.

	NOX■[13]	NADPH oxidases regulate endothelial inflammatory injury induced by $PM_{2.5}$ via AKT/eNOS/NO axis
	AMPK▲[14]	AMPK plays a role in $PM_{2.5}$-induced lung injury and cardiac dysfunction
	NLRP3 ■[15]	Crosstalk between macrophage polarization and NLRP3 inflammasome activation mediates $PM_{2.5}$-induced cardiac injury.
Nerve	PI3K/Akt/FoxO1▲[16]	Activation of the PI3K/AKT/FoxO1 pathway is associated with $PM_{2.5}$-induced brain injury
	JAK2/STAT3; MAPK; NF-κB▲+■[17]	NF-κB and Nrf2 signaling play a role in $PM_{2.5}$-induced metabolic disorders and neuroinflammation.
	NLRP3■[18]	$PM_{2.5}$ promotes AD pathogenesis via NLRP3 inflammasome-mediated neuroinflammation
	AMPK/mTOR■[19]	The AMPK/mTOR signaling pathway plays a key role in PM2.5-induced brain diseases.
Immunity	Nrf2▲[20]	During $PM_{2.5}$ exposure, NRF2 participates in stress-induced myelopoiesis, aggravates lung injury, and leads to systemic inflammation.
	NF-κB ▲[21]	$PM_{2.5}$ may affect SLE through involvement of the NF-κB pathway.
	Endoplasmic reticulum stress▲[22]	$PM_{2.5}$ exposure mediated immunotoxicity in SD rats through the stimulation of endoplasmic reticulum stress (ERS) and autophagy.
	PI3K/AKT/mTOR▲[23]	$PM_{2.5}$ induces alveolar macrophage autophagy through oxidative stress-mediated PI3K/AKT/mTOR pathway.
	Immune imbalance▲[24]	IL-6, DEC205, and CD86 serve as predictors of $PM_{2.5}$-induced immune effects, leading to inflammation and immune responses
Endocrine	Nrf2 ▲[25]	$PM_{2.5}$ induces hepatic insulin resistance through Nrf2-

		mediated oxidative stress and JNK-mediated signaling.
	NF-κB▲+■[26]	$PM_{2.5}$-induced adiposity and insulin resistance are associated with NF-κB-mediated oxidative stress pathways.
Digestion	Nrf2/SIKE▲+■[27]	Nrf2/SIKE may be involved in $PM_{2.5}$-induced liver injury
	Nrf2/JNK▲[28]	$PM_{2.5}$ induces oxidative stress and inflammatory responses via the Nrf2/JNK pathway.
	NLRP3▲[29]	$PM_{2.5}$ may exacerbate acute liver injury by promoting NLRP3-mediated M1 polarization in Kupffer cells and creating an inflammatory microenvironment.
	TLR4/Myd88/NF-κB ▲[30]	The TLR4/MyD88 signaling pathway may play a role in $PM_{2.5}$-induced liver injury upon activation.
	PINK1/Parkin/LC3■[31]	$PM_{2.5}$ induces mitophagy and subsequent liver fibrosis by activating the PINK1/Parkin pathway through ROS.
	Disruption of intestinal microbiota▲[32]	$PM_{2.5}$ induces gastrointestinal diseases through gut microbiota disruption.
Genitourinary	PI3K/Akt▲[33]	PM2.5 induces reproductive impairment in male rats via PI3K/Akt overexpression
	NF-κB/COX-2/PGE2 ▲[34]	$PM_{2.5}$ induces oxidative stress damage through activation of the NF-κB/COX-2/PGE2 signaling pathway.
	NALP3▲[35]	$PM_{2.5}$ impairs sperm quality by mediating the NALP3 inflammasome and the miR-183/96/182 cluster, which target FOXO1.
	Endoplasmic reticulum stress▲[36]	$PM_{2.5}$ exposure induced reproductive toxicity in male SD rats by the stimulation of ERS.
	NF-κB▲[37]	$PM_{2.5}$ affects the ovarian reserve in offspring by activating the PI3K/AKT/FoxO3a pathway and the ROS-dependent NF-κB pathway.

UPR/JNK▲[38]	UPR-mediated JNK pathway mediates $PM_{2.5}$-induced testicular cell apoptosis in offspring.
MAPK ▲[39]	$PM_{2.5}$ impairs spermatogenesis through ROS-mediated MAPK signaling.
IRE1/JNK▲[40]	$PM_{2.5}$ exposure induces reproductive injury through IRE1/JNK/autophagy signaling in male rats
TGF-β3/p38▲[41]	$PM_{2.5}$ disrupts the blood-brain barrier through activation of the TGF-β3/p38 MAPK pathway.

1. Dou, C. M.; Zhang, J.; Qi, C. C., Cooking oil fume-derived PM2.5 induces apoptosis in A549 cells and MAPK/NF-kappa B/STAT1 pathway activation. *Environ Sci Pollut Res Int* **2018,** *25* (10), 9940-9948.
2. Han, X. M.; Zhuang, Y., PM2.5 induces autophagy-mediated cell apoptosis via PI3K/AKT/mTOR signaling pathway in mice bronchial epithelium cells. *Exp Ther Med* **2021,** *21* (1), 1.
3. Xu, X.; Wang, H.; Liu, S.; Xing, C.; Liu, Y.; Aodengqimuge; Zhou, W.; Yuan, X.; Ma, Y.; Hu, M.; Hu, Y.; Zou, S.; Gu, Y.; Peng, S.; Yuan, S.; Li, W.; Ma, Y.; Song, L., TP53-dependent autophagy links the ATR-CHEK1 axis activation to proinflammatory VEGFA production in human bronchial epithelial cells exposed to fine particulate matter (PM2.5). *Autophagy* **2016,** *12* (10), 1832-1848.
4. Jia, Y.; Li, X.; Nan, A.; Zhang, N.; Chen, L.; Zhou, H.; Zhang, H.; Qiu, M.; Zhu, J.; Ling, Y.; Jiang, Y., Circular RNA 406961 interacts with ILF2 to regulate PM2.5-induced inflammatory responses in human bronchial epithelial cells via activation of STAT3/JNK pathways. *Environ Int* **2020,** *141*, 105755.
5. Zhu, X., M, ; Wang, Q.; Xing, W., W,; Long, M., H, ; Fu, W., L, ; Xia, W., R, ; Jin, C.; Guo, N.; Xu, D., Q, ; Xu, D., G. , PM2.5 induces autophagy-mediated cell death via NOS2 signaling in human bronchial epithelium cells. *Int J Biol Sci* **2018,** *14* (5), 557-564.
6. Pu, X., J, ; Li, J.; Zhou, Q., L, ; Pan, W.; Li, Y., Q, ; Zhang, Y.; Wang, J.; Jiao, Z., Rosiglitazone inhibits PM2.5-induced cytotoxicity in human lung epithelial A549 cells. *Ann Transl Med* **2018,** *6* (8), 152.
7. Wang, C.; Meng, X.; Meng, M.; Shi, M.; Sun, W.; Li, X.; Zhang, X.; Liu, R.; Fu, Y.; Song, L., Oxidative stress activates the TRPM2-Ca2+-NLRP3 axis to promote PM2.5-induced lung injury of mice. *Biomedicine & pharmacotherapy = Biomedecine & pharmacotherapie* **2020,** *130* (2020), 110481.
8. Wang, Q.; Gan, X.; Li, F.; Chen, Y.; Fu, W.; Zhu, X.; Xu, D.; Long, M.; Xu, D., PM(2.5) Exposure Induces More Serious Apoptosis of Cardiomyocytes Mediated by Caspase3 through JNK/ P53 Pathway in Hyperlipidemic Rats. *Int J Biol Sci* **2019,** *15* (1), 24-33.
9. Feng, L.; Wei J; Liang S; Sun Z; J., D., miR-205/IRAK2 signaling pathway is associated

with urban airborne PM2.5-induced myocardial toxicity. *Nanotoxicology* **2020,** *14* (9), 1198-1212.
10. Meng, M. L.; Jia, R. X.; Wei, M.; Meng, X. Z.; Zhang, X.; Du, R.; Sun, W. P.; Wang, L. L.; Song, L. Y., Oxidative stress activates Ryr2-Ca2+ and apoptosis to promote PM2.5-induced heart injury of hyperlipidemia mice. *Ecotoxicology and Environmental Safety* **2022,** *232*.
11. Yin, J.; Xia, W.; Li, Y.; Guo, C.; Zhang, Y.; Huang, S.; Jia, Z.; Zhang, A., COX-2 mediates PM2.5-induced apoptosis and inflammation in vascular endothelial cells. *Am J Transl Res* **2017,** *9* (9), 3967-3976.
12. Gao, M.; Ma, Y.; Luo, J.; Li, D.; Jiang, M.; Jiang, Q.; Pi, J.; Chen, R.; Chen, W.; Zhang, R.; Zheng, Y.; Cui, L., The Role of Nrf2 in the PM-Induced Vascular Injury Under Real Ambient Particulate Matter Exposure in C57/B6 Mice. *Frontiers in pharmacology* **2021,** *12*, 618023.
13. Zou, L.; Xiong, L.; Wu, T.; Wei, T.; Liu, N.; Bai, C.; Huang, X.; Hu, Y.; Xue, Y.; Zhang, T.; Tang, M., NADPH oxidases regulate endothelial inflammatory injury induced by PM(2.5) via AKT/eNOS/NO axis. *J Appl Toxicol* **2022,** *42* (5), 738-749.
14. Wang, H.; Shen, X.; Tian, G.; Shi, X.; Huang, W.; Wu, Y.; Sun, L.; Peng, C.; Liu, S.; Huang, Y.; Chen, X.; Zhang, F.; Chen, Y.; Ding, W.; Lu, Z., AMPKα2 deficiency exacerbates long-term PM(2.5) exposure-induced lung injury and cardiac dysfunction. *Free Radic Biol Med* **2018,** *121*, 202-214.
15. Du, X.; Jiang, S.; Zeng, X.; Zhang, J.; Pan, K.; Song, L.; Zhou, J.; Kan, H.; Sun, Q.; Zhao, J.; Xie, Y., Fine particulate matter-induced cardiovascular injury is associated with NLRP3 inflammasome activation in Apo E(-/-) mice. *Ecotoxicol Environ Saf* **2019,** *174*, 92-99.
16. Song, L.; Pan, K.; Du, X.; Jiang, S.; Zeng, X.; Zhang, J.; Lei, L.; Zhang, M.; Zhang, Y.; Fan, D.; Liu, Z.; Zhou, J.; Zhao, J., Ambient PM(2.5)-induced brain injury is associated with the activation of PI3K/AKT/FoxO1 pathway. *Environ Sci Pollut Res Int* **2021,** *28* (48), 68276-68287.
17. Xu, M. X.; Zhu, Y. F.; Chang, H. F.; Liang, Y., Nanoceria restrains PM2.5-induced metabolic disorder and hypothalamus inflammation by inhibition of astrocytes activation related NF-κB pathway in Nrf2 deficient mice. *Free Radic Biol Med* **2016,** *99*, 259-272.
18. Shi, J. Q.; Wang, B. R.; Jiang, T.; Gao, L.; Zhang, Y. D.; Xu, J., NLRP3 Inflammasome: A Potential Therapeutic Target in Fine Particulate Matter-Induced Neuroinflammation in Alzheimer's Disease. *J Alzheimers Dis* **2020,** *77* (3), 923-934.
19. Ren, F.; Xu, X.; Xu, J.; Mei, Y.; Zhang, J.; Wang, X.; Li, F., Compound essential oils relieve oxidative stress caused by PM(2) (.5) exposure by inhibiting autophagy through the AMPK/mTOR pathway. *Environ Toxicol* **2021,** *36* (9), 1765-1774.
20. Wang, Y.; Jin, X.; Li, M.; Gao, J.; Zhao, X.; Ma, J.; Shi, C.; He, B.; Hu, L.; Shi, J.; Liu, G.; Qu, G.; Zheng, Y.; Jiang, G., PM(2.5) Increases Systemic Inflammatory Cells and Associated Disease Risks by Inducing NRF2-Dependent Myeloid-Biased Hematopoiesis in Adult Male Mice. *Environ Sci Technol* **2023,** *57* (21), 7924-7937.
21. Yariwake VY; Torres JI; Dos Santos ARP; Freitas SCF; De Angelis K; Farhat SCL; Câmara NOS; MM., V., Chronic exposure to PM2.5 aggravates SLE manifestations in lupus-prone mice. *Part Fibre Toxicol* **2021,** *18* (1), 15.
22. Su, R.; Jin, X.; Lyu, L.; Tian, J.; Amin, S.; Li, Z., The potential immunotoxicity of fine particulate matter based on SD rat spleen. *Environ Sci Pollut Res Int* **2019,** *26* (23), 23958-23966.
23. Su, R.; Jin, X.; Zhang, W.; Li, Z.; Liu, X.; Ren, J., Particulate matter exposure induces the autophagy of macrophages via oxidative stress-mediated PI3K/AKT/mTOR pathway.

Chemosphere **2017,** *167*, 444-453.
24. Honda A; Fukushima W; Oishi M; Tsuji K; Sawahara T; Hayashi T; Kudo H; Kashima Y; Takahashi K; Sasaki H; Ueda K; H., T., Effects of Components of PM2.5 Collected in Japan on the Respiratory and Immune Systems. *Int J Toxicol* **2017,** *36* (2), 153-164.
25. Xu J; Zhang W; Lu Z; Zhang F; W., D., Airborne PM2.5-Induced Hepatic Insulin Resistance by Nrf2/JNK-Mediated Signaling Pathway. *Int J Environ Res Public Health* **2017,** *14* (7), 1-15.
26. Wang, N.; Ma, Y.; Liu, Z.; Liu, L.; Yang, K.; Wei, Y.; Liu, Y.; Chen, X.; Sun, X.; Wen, D., Hydroxytyrosol prevents PM2.5-induced adiposity and insulin resistance by restraining oxidative stress related NF-kappa B pathway and modulation of gut microbiota in a murine model. *Free Radic Biol Med* **2019,** *141*, 393-407.
27. Ge, C. X.; Tan, J.; Zhong, S. Y.; Lai, L. L.; Chen, G.; Zhao, J. J.; Yi, C.; Wang, L. Y.; Zhou, L. W.; Tang, T. T.; Yang, Q. F.; Lou, D. S.; Li, Q.; Wu, Y. K.; Hu, L. F.; Kuang, G.; Liu, X.; Wang, B. C.; Xu, M. X., Nrf2 mitigates prolonged PM2.5 exposure-triggered liver inflammation by positively regulating SIKE activity: Protection by Juglanin. *Redox Biology* **2020,** *36*, 101645.
28. Zhang, Q.; Zhang, H. Y.; Yu, X. Q.; Cui, Z. J.; Lv, Z. W., Impact of particulate matter 2.5 on the liver function of mice. *European review for medical and pharmacological sciences* **2023,** *27* (10), 4357-4368.
29. Pei, H.; He, Z.; Du, R.; Zhu, Y.; Yang, Y., PM2.5 exposure aggravates acute liver injury by creating an inflammatory microenvironment through Kupffer cell. *Ecotoxicol Environ Saf* **2023,** *263*, 115264.
30. Ya, P.; Xu, H.; Ma, Y.; Fang, M.; Yan, X.; Zhou, J.; Li, F., Liver injury induced in Balb/c mice by PM(2.5) exposure and its alleviation by compound essential oils. *Biomedicine & pharmacotherapy = Biomedecine & pharmacotherapie* **2018,** *105*, 590-598.
31. Qiu, Y. N.; Wang, G. H.; Zhou, F.; Hao, J. J.; Tian, L.; Guan, L. F.; Geng, X. K.; Ding, Y. C.; Wu, H. W.; Zhang, K. Z., PM2.5 induces liver fibrosis via triggering ROS-mediated mitophagy. *Ecotoxicol Environ Saf* **2019,** *167*, 178-187.
32. Zhang, Y.; Li, M.; Pu, Z.; Chi, X.; Yang, J., Multi-omics data reveals the disturbance of glycerophospholipid metabolism and linoleic acid metabolism caused by disordered gut microbiota in PM2.5 gastrointestinal exposed rats. *Ecotoxicol Environ Saf* **2023,** *262*, 115182.
33. Cao, X., N, ; Yan, C.; Liu, D., Y, ; Peng, J., P, ; Chen, J., J, ; Zhou, Y.; Long, C., L, ; He, D., W, ; Lin, T.; Shen, L., J, ; Wei, G., H., Fine particulate matter leads to reproductive impairment in male rats by overexpressing phosphatidylinositol 3-kinase (PI3K)/protein kinase B (Akt) signaling pathway. *Toxicol Lett* **2015,** *237* (3), 181-190.
34. Liu, J., N, ; Zheng, J.; Yin, Y., J,; Zhang, Z., H,; Sheng, X.; Tang, W., The mechanism of PM 2.5 exposure-induced activation of NF-kB/COX-2/PGE2 signaling pathway in oxidative stress damage to male reproductive function. *Practical Journal of Clinical Medicine* **2021,** *18* (5), 9-13.
35. Zhou, L.; Su, X.; Li, B.; Chu, C.; Sun, H.; Zhang, N.; Han, B.; Li, C.; Zou, B.; Niu, Y.; Zhang, R., PM2.5 exposure impairs sperm quality through testicular damage dependent on NALP3 inflammasome and miR-183/96/182 cluster targeting FOXO1 in mouse. *Ecotoxicol Environ Saf* **2019,** *169*, 551-563.
36. Liu, X.; Jin, X.; Su, R.; Li, Z., The reproductive toxicology of male SD rats after PM(2.5)

exposure mediated by the stimulation of endoplasmic reticulum stress. *Chemosphere* **2017,** *189*, 547-555.
37. Chen, Y.; Xi, Y.; Li, M.; Wu, Y.; Yan, W.; Dai, J.; Wu, M.; Ding, W.; Zhang, J.; Zhang, F.; Zhou, S.; Wang, S., Maternal exposure to PM2.5 decreases ovarian reserve in neonatal offspring mice through activating PI3K/AKT/FoxO3a pathway and ROS-dependent NF-κB pathway. *Toxicology* **2022,** *481*, 153352.
38. Ren, L.; Jiang, J.; Huang, J.; Zang, Y.; Huang, Q.; Zhang, L.; Wei, J.; Lu, H.; Wu, S.; Zhou, X., Maternal exposure to PM2.5 induces the testicular cell apoptosis in offspring triggered by the UPR-mediated JNK pathway. *Toxicol Res (Camb)* **2022,** *11* (1), 226-234.
39. Liu, B.; Wu, S. D.; Shen, L. J.; Zhao, T. X.; Wei, Y.; Tang, X. L.; Long, C. L.; Zhou, Y.; He, D. W.; Lin, T.; Wei, G. H., Spermatogenesis dysfunction induced by PM2.5 from automobile exhaust via the ROS-mediated MAPK signaling pathway. *Ecotoxicology and Environmental Safety* **2019,** *167*, 161-168.
40. Yang, Y.; Feng, Y.; Huang, H.; Cui, L.; Li, F., PM2.5 exposure induces reproductive injury through IRE1/JNK/autophagy signaling in male rats. *Ecotoxicol Environ Saf* **2021,** *211*, 111924.
41. Liu, J.; Ren, L.; Wei, J.; Zhang, J.; Zhu, Y.; Li, X.; Jing, L.; Duan, J.; Zhou, X.; Sun, Z., Fine particle matter disrupts the blood-testis barrier by activating TGF-β3/p38 MAPK pathway and decreasing testosterone secretion in rat. *Environ Toxicol* **2018,** *33* (7), 711-719.
