# Supplementary material for: Health Risk and Pathogenesis of PM2.5 in Human Systems
Source: Toxics. 2026 Mar 27;14(4):286. doi: 10.3390/toxics14040286 (PMC13120000; doi:10.3390/toxics14040286)
Supplement: Supplementary file 1 [file toxics-14-00286-s001.zip › Table S4.pdf]

Table S4. Summary of literature search and screening by organ system

System	Records identified	After deduplication	After title/abstract screening	Included in review(actual)
Respiratory system	8537	7389	2463	44
Cardiovascular system	5057	2069	773	41
Nervous system	1497	1335	432	35
Immune system	667	642	239	15
Endocrine system	2181	1092	328	21
Digestive system	739	691	140	18
Genitourinary System	309	296	100	34

Note: Given the nature of this narrative review, the numbers presented in the table are semi-quantitative estimates intended to illustrate the overall screening process. The data in Table S4 are derived from the sampling process described above, while the actual numbers of references cited from each system (also listed in the table) reflect the outcomes of full-text review, content evaluation, and cross-system deduplication. Any discrepancy between the two is expected for a narrative review and does not compromise the transparency of the screening process. Additionally, Baidu Scholar and Google Scholar were used as supplementary sources; their results largely overlapped with those from the core databases and were therefore not counted separately.
